# Functional Knowledge Transfer for High-accuracy Prediction of Under-studied Biological Processes

**DOI:** 10.1371/journal.pcbi.1002957

**Published:** 2013-03-14

**Authors:** Christopher Y. Park, Aaron K. Wong, Casey S. Greene, Jessica Rowland, Yuanfang Guan, Lars A. Bongo, Rebecca D. Burdine, Olga G. Troyanskaya

**Affiliations:** 1 Department of Computer Science, Princeton University, Princeton, New Jersey, United States of America; 2 Lewis-Sigler Institute for Integrative Genomics, Princeton University, Princeton, New Jersey, United States of America; 3 Department of Molecular Biology, Princeton University, Princeton, New Jersey, United States of America; 4 Department of Computational Medicine and Bioinformatics, University of Michigan, Ann Arbor, Michigan, United States of America; 5 Department of Computer Science, University of Tromsø, Tromsø, Norway; National University of Singapore, Singapore

## Abstract

A key challenge in genetics is identifying the functional roles of genes in pathways. Numerous functional genomics techniques (e.g. machine learning) that predict protein function have been developed to address this question. These methods generally build from existing annotations of genes to pathways and thus are often unable to identify additional genes participating in processes that are not already well studied. Many of these processes are well studied in *some* organism, but not necessarily in an investigator's organism of interest. Sequence-based search methods (e.g. BLAST) have been used to transfer such annotation information between organisms. We demonstrate that functional genomics can complement traditional sequence similarity to improve the transfer of gene annotations between organisms. Our method transfers annotations only when functionally appropriate as determined by genomic data and can be used with any prediction algorithm to combine transferred gene function knowledge with organism-specific high-throughput data to enable accurate function prediction.

We show that diverse state-of-art machine learning algorithms leveraging functional knowledge transfer (FKT) dramatically improve their accuracy in predicting gene-pathway membership, particularly for processes with little experimental knowledge in an organism. We also show that our method compares favorably to annotation transfer by sequence similarity. Next, we deploy FKT with state-of-the-art SVM classifier to predict novel genes to 11,000 biological processes across six diverse organisms and expand the coverage of accurate function predictions to processes that are often ignored because of a dearth of annotated genes in an organism. Finally, we perform *in vivo* experimental investigation in *Danio rerio* and confirm the regulatory role of our top predicted novel gene, *wnt5b*, in leftward cell migration during heart development. FKT is immediately applicable to many bioinformatics techniques and will help biologists systematically integrate prior knowledge from diverse systems to direct targeted experiments in their organism of study.

## Author Summary

Due to technical and ethical challenges many human diseases or biological processes are studied in model organisms. Discoveries in these organisms are then transferred back to human or other model organisms. Traditional methods for transferring novel gene function annotations have relied on finding genes with high sequence similarity believed to share evolutionary ancestry. However, sequence similarity does not guarantee a shared functional role in molecular pathways. In this study, we show that functional genomics can complement traditional sequence similarity measures to improve the transfer of gene annotations between organisms. We coupled our knowledge transfer method with current state-of-the-art machine learning algorithms and predicted gene function for 11,000 biological processes across six organisms. We experimentally validated our prediction of *wnt5b*'s involvement in the determination of left-right heart asymmetry in zebrafish. Our results show that functional knowledge transfer can improve the coverage and accuracy of machine learning methods used for gene function prediction in a diverse set of organisms. Such an approach can be applied to additional organisms, and will be especially beneficial in organisms that have high-throughput genomic data with sparse annotations.

## Introduction

Defining the role of proteins in pathways is among the key challenges of human genomics. Many successful approaches have been developed for prediction of protein function and pathway membership [1]–[6], however they rely on prior knowledge in the organism of interest to make new predictions (i.e. at least some genes in the organism already annotated to the pathway) [7]–[11]. These approaches rely on identifying characteristic behavioral patterns, in functional genomic datasets, phylogenetic profiles, or genomic feature studies of genes that are known to participate in a pathway, then use these patterns to predict additional pathway members [12]–[14]. For example, gene expression and protein interaction profiles can be used by machine learning methods to associate novel genes to pathways based on previously known pathway members [15], [16]. The potential of such computational approaches to direct experiments has been demonstrated in studies investigating mitochondrial biogenesis [17] and seed pigmentation [18]. Other common exploratory methods, such as hierarchical clustering [19], don't directly use known gene annotations to learn a prediction classifier, however they often use existing annotations to interpret the resulting cluster of genes (e.g. gene enrichment analysis) [20]. However in many organisms including human, pathways and processes where functional annotations of genes are most needed often have few or no prior experimentally confirmed annotations, making novel predictions of genes that may participate in such a process difficult or impossible. Thus, our study describes a method to robustly increase the set of prior gene annotations, which has the potential to improve all function prediction methods by increasing the accuracy of their predictions and enabling wider coverage of pathways and biological processes.

**Figure 1:**
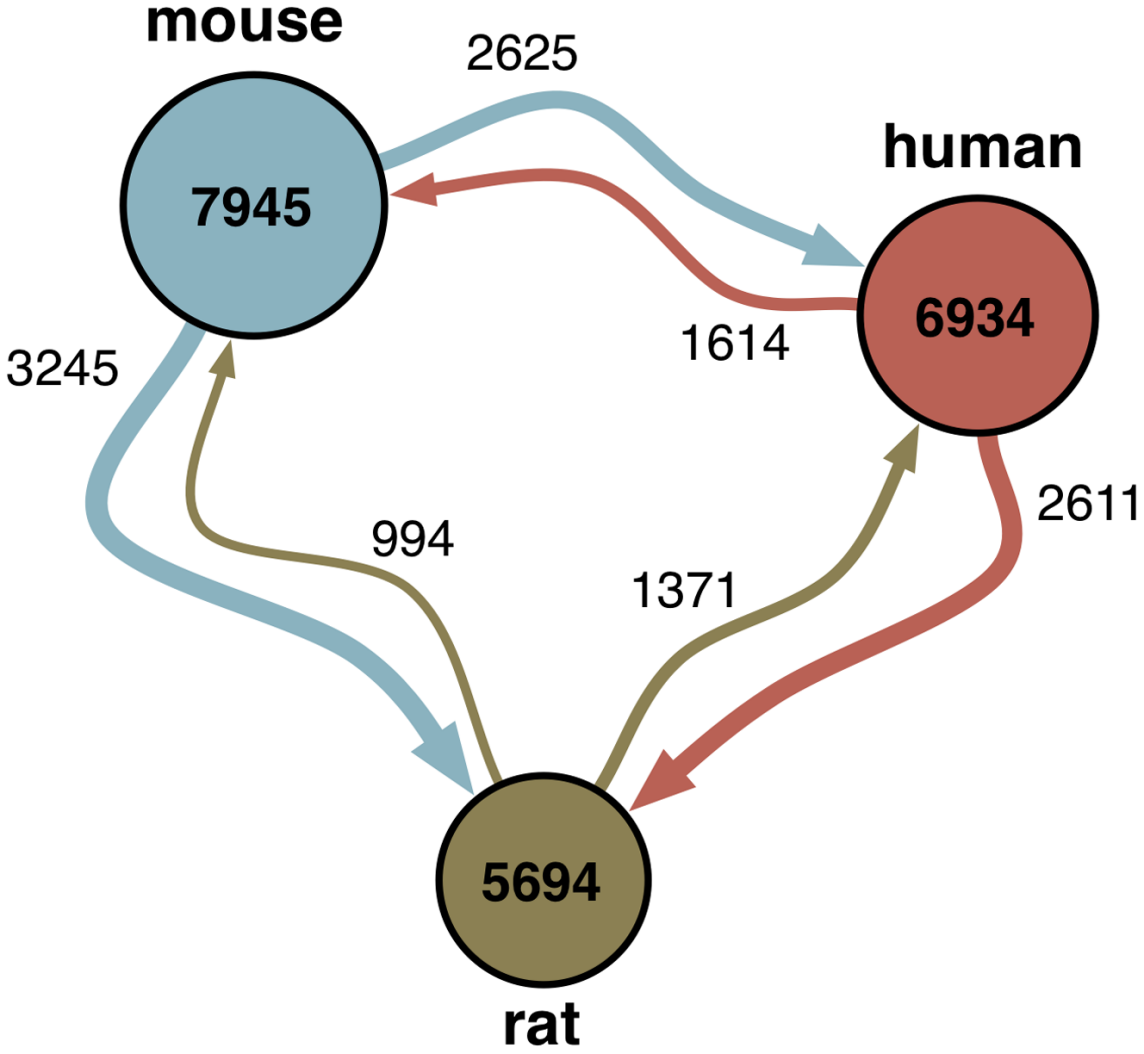
Functional knowledge of biological processes is far from uniform, even among closely related organisms. Each node represents the number of experimentally annotated biological processes in an organism. Each edge value corresponds to the number of experimentally annotated processes in the source organism that lack any experimental annotations in the target organism. Thus, the directed edges between nodes indicate the direction of potential annotation transfer between organisms. For example, 3,245 processes with annotations in mouse have no experimentally annotated genes in rat.

Many of these processes are well studied in *some* model organism, but not necessarily in an investigator's organism of interest. Even when applying a conservative examination of only the closely related and heavily studied mammalian species human, mouse, and rat, processes represented in one species are often not well-characterized in another (summarized in Figure 1 and a full list of processes available in Text S1). For example, the process *cellular glucose homeostasis*, an increasingly important process due to the role of cellular metabolism in cancer development, has less than 5 gene annotations in human, yet has 31 in mouse, a commonly used model organism for cancer studies. These processes (referred to in the text as *understudied processes*) are not well studied in a particular organism of interest (i.e. very few genes are annotated) but might be well characterized in some other organism.

A longstanding solution to improving the prediction accuracy of understudied processes has been to transfer functional annotations from organisms where the process is better characterized [21]. The critical challenge in accurately transferring functional knowledge between organisms is identifying the appropriate genes for the transfer: those genes that share not only sequence similarity, but also conserved pathway roles. Large-scale automated methods have so far exclusively used sequence homology to identify functionally conserved genes [22], [23]. However, the relationship between sequence similarity and function is not trivial. For example, human angiopoietin-4 (ANGPT4), an important angiogenesis growth factor, has been shown to activate TEK (tyrosine-protein kinase receptor), while the mouse sequence-ortholog (Angpt4) has been shown to inhibit TEK [24].

**Figure 2:**
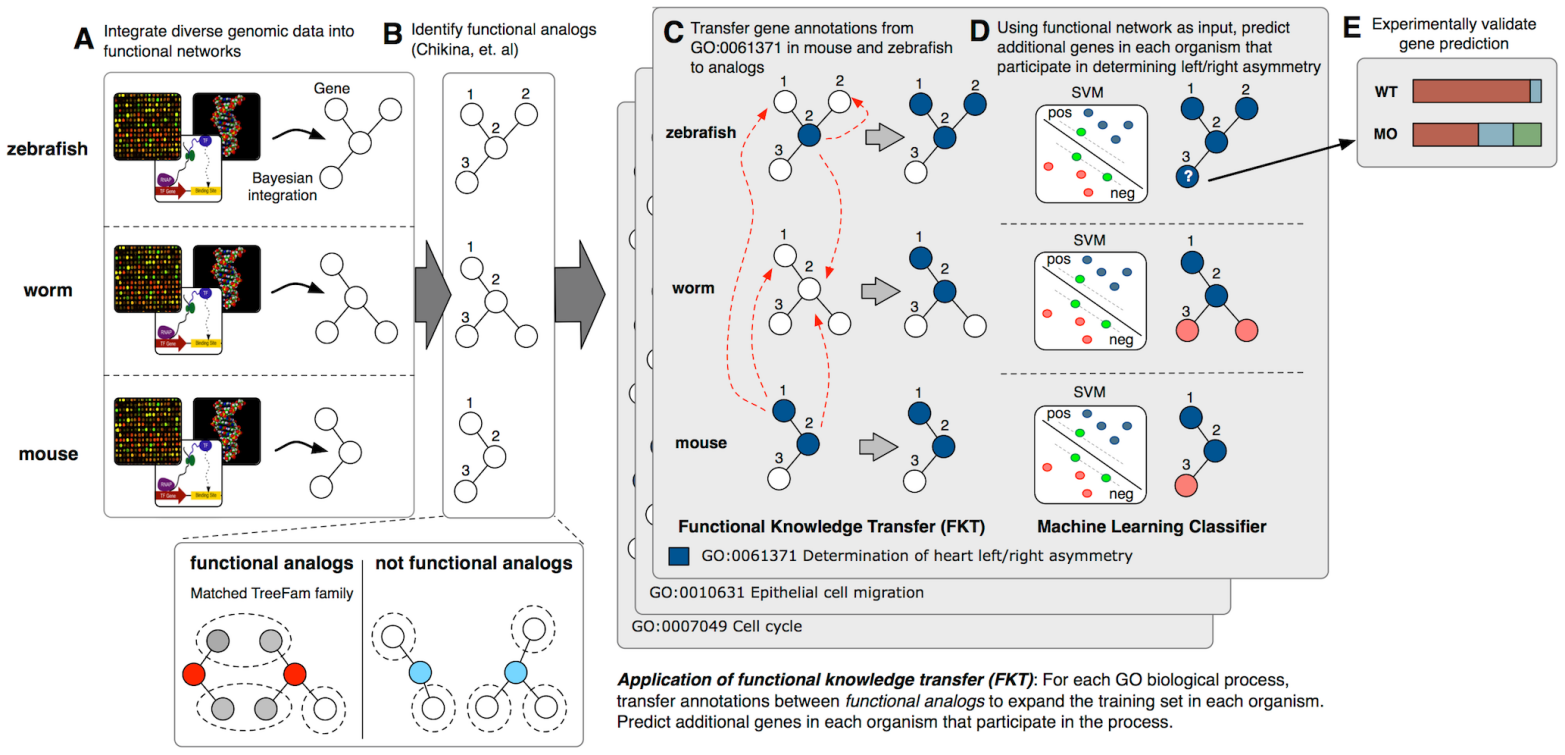
Schematic of the functional knowledge transfer. (A) A functional relationship network is constructed for each organism through Bayesian integration of heterogeneous genomic data (e.g. expression, TF motif binding, physical interaction assays). (B) Functionally analogous gene pairs (i.e. functional analogs) are identified by computing a gene pairwise functional similarity score introduced in Chikina et. al between all sequence homologs. Functional similarity is measured by the statistical significance of the number of common TreeFam gene families in the functional relationship network neighbors of each homologous gene pair. (C) Next, experimentally confirmed biological process annotations for each gene are transferred to its functional analogs. (D) For each biological process the extended set of gene annotations (which include direct gene annotations, if available, and cross-annotated genes) can be used as training examples for machine learning methods (SVM used in this study) to make novel gene membership predictions. (E) Top predicted genes are carried over for experimental validation.

In our previous work [25], we developed a cross-organism gene functional similarity measure, which relied on the concept that functional genomics data can be used to resolve homologous relationships among closely related genes. The approach summarizes the compendium of genomics data in each organism into functional relationship networks to identify genes that do not simply share sequence similarity but also functional behavior in large collections of heterogeneous functional data, and are thus functionally analogous (referred to in text as *functional analogs*). In this current study, we present a novel knowledge transfer method, Functional Knowledge Transfer (also referred to in text as FKT and outlined in Figure 2), which leverages the mapping of functional analogs to direct cross-organism annotation transfer for function prediction. FKT can be especially beneficial for existing and future machine learning methods studying biological processes with sparse annotations in any given organism of interest. By transferring experimental knowledge between genes that have been identified as functional analogs, our method extends beyond simple annotation transfer by sequence similarity. Experimental functional annotations are only transferred for genes that are not just similar in sequence, but also in their functional behavior derived from a large and relatively comprehensive compendium of genomic data.

In this study, we show that FKT improves the prediction accuracy of machine learning algorithms, particularly for biological processes with few existing annotations in an organism of study. We compare FKT to annotation transfer by sequence similarity (BLAST) and demonstrate the superior performance of our method in improving gene function prediction performance. The consistent improvement and high performance across various state-of-the-art classification algorithms demonstrates our approach is robust to different learning models, which is crucial for wide applicability.

We apply FKT to gene function (i.e. biological process) prediction in six metazoan organisms (*Homo sapiens*, *Mus musculus*, *Rattus novegicus*, *Drosophila melanogaster*, *Danio rerio* and *Caenorhabditis elegans*) and show that FKT is robust enough for the automated transfer of annotations among these diverse organisms and accurate function prediction. Finally, we demonstrate an application of FKT to discovering novel biology by coupling the knowledge transfer with a Support Vector Machine (SVM) to predict proteins involved in left-right asymmetry regulation during heart development in *Danio rerio*. We correctly predict several proteins in the pathway and experimentally confirm the first evidence of *wnt5b*'s role in the process. A comprehensive application of FKT to 11,000 biological processes, along with the functional relationship networks for all six organisms, are available through the IMP web-server portal accessible at http://imp.princeton.edu [26].

## Results

In Figure 2, we outline the pipeline for FKT and the subsequent gene function predictions (details provided in the Materials and Methods section below). Briefly, we first integrated high and low-throughput experimental data such as gene expression data, protein-protein interaction data, protein domain and transcription factor binding motif information into functional networks for each of seven organisms (*Saccharomyces cerevisiae* was also included as an annotation source). Next, we calculated a network-based functional similarity score as described in our prior work [25] but extended here to additional organisms and data sources, between all ortholog and paralog pairs in a Treefam [22] gene family to identify the targets for annotation transfer. Homologs with high functional similarity scores were determined to be functional analogs. Next, we applied FKT by transferring all gene-process annotations between functional analogs and merge these with existing annotations (if available) in an organism. To test the predictive power of FKT, the set of transferred and organism-specific annotations were used to train a Support Vector Machine (SVM) classifier [27] and predict new genes to all biological processes in six metazoan organisms. Functional network connection weights (i.e. the inferred probability that two genes co-function in the same biological process), were treated as input features to the classifier (see Materials and Methods). Additional state-of-art machine learning methods (L1-regularized logistic regression [28] and Random forest [29]) were trained and evaluated to test the robustness of FKT performance improvement. Finally, we demonstrate the power of our approach with an *in vivo* experiment validating the predicted role of wnt5b in establishing correct heart asymmetry in *Danio rerio*.

### Functional knowledge transfer enables accurate gene prediction for pathways with few or no known genes

Most modern machine-learning methods that predict novel members of a biological pathway require a set of genes already known to participate in the pathway. These approaches are therefore limited to predicting genes to biological processes with sufficient prior knowledge in an organism [30]. For example, in the MouseFunc competition [7] (a broad competition focused on the performance of biological process prediction approaches), terms with less than three gene annotations were considered infeasible to predict and not included.

**Figure 3:**
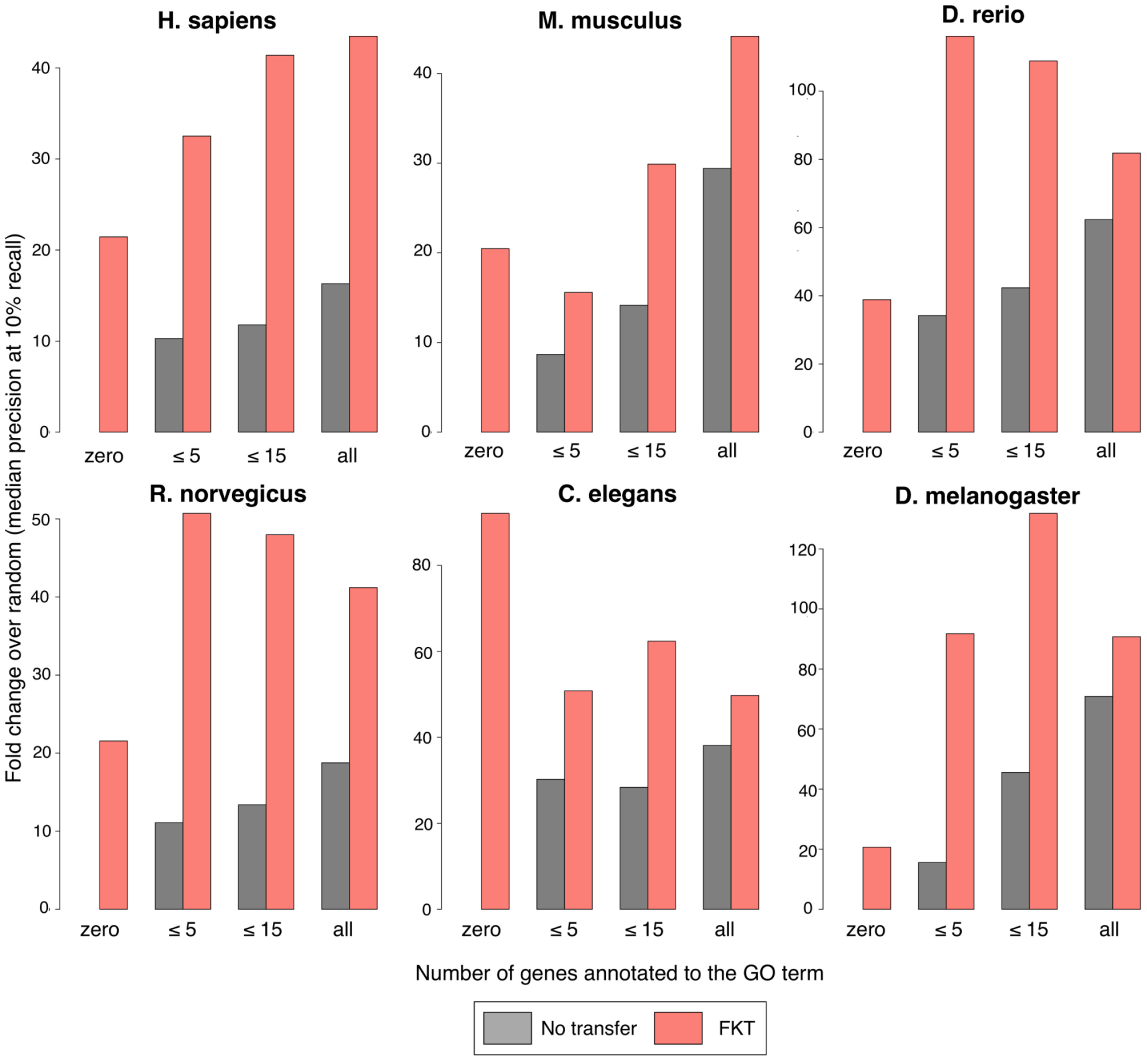
Cross annotation allows accurate recovery of small and unannotated terms. All annotations accumulated after 5/11/2008 are held out from our prediction pipeline (as outlined in Figure 2) and are used for evaluation of prediction performance. 3,207 GO biological processes terms that acquired new annotations subsequent to our holdout date are grouped by organism and by the number of annotations at 5/11/2008 (zero, < = 5, < = 15, all). Performances at recapitulating future annotations are compared for a machine learning method (SVM) without (gray) and with (pink) including functional knowledge transfer (FKT) derived examples. For processes with zero annotations before 5/11/2008, no predictions can be made without cross-annotation (shown as absent performance bar). In all six metazoan organisms and for all process sizes, FKT improves prediction performance.

We address this constraint by leveraging knowledge across species, which allows us to take advantage of known biology from a model organism where the pathway of interest may be better studied. We applied our functional cross-annotation strategy (FKT) to biological processes with few known genes (annotation sizes of < = 5 and < = 15) in six metazoans and evaluated the predictive performance of an SVM trained with these annotations. To evaluate our performance, we constructed a three-year temporal holdout of experimental annotations. We used only biological process annotations added to Gene Ontology [31] before 5/11/2008 (all dates in mm/dd/yyyy format) in learning the functional networks, transferring annotations across organisms, and predicting gene-process participation. New experimental annotations added to Gene Ontology between 5/11/2008 to 5/11/2011 were held-out and used for evaluation. In total, 3,207 GO biological process terms across the six organisms acquired new gene annotations in the subsequent three years. We evaluated the accuracy of our predictions with the gene-process assignments made during the hold-out time period in Figure 3 (evaluation results of all GO terms in Text S3). We observed substantial improvement using FKT when compared with only using the direct annotations for an organism. Improvement was evident across all six organisms, suggesting that even well characterized model organisms (e.g. mouse) can benefit from genomic-data-driven knowledge transfer. In addition, by holding out gene-process annotations acquired within the last three years, we could evaluate our ability to predict genes to processes which had no known genes in an organism prior to the hold out date (i.e. before 5/11/2008). Even though these processes were uncharacterized at that time, they subsequently became the focus of a directed experiment and thus were deemed biologically relevant and experimentally feasible in the organism. As shown in Figure 3, FKT gene predictions to these processes performed competitively even compared to biological processes with known gene annotations. Furthermore, these results were robust to the evaluation timeframe (1-year temporal holdout shown in Figure S1).

**Figure 4:**
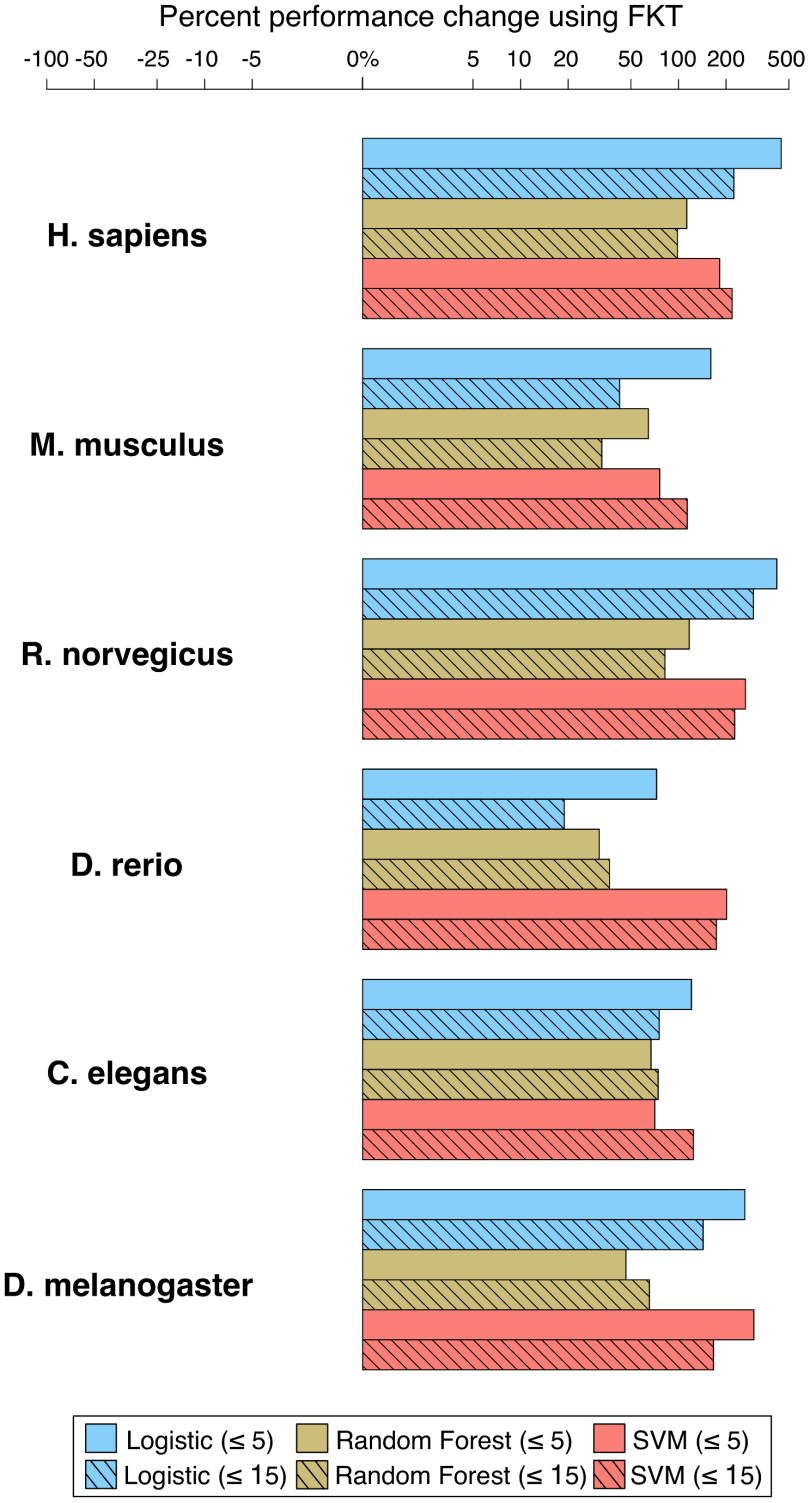
Functional knowledge transfer (FKT) improves prediction accuracy for a wide range of state-of-the-art classification algorithms. The performance change when applying FKT are compared for each of three machine learning algorithms: L1-regularized logistic regression, Random forest and SVM (evaluated based on the ability to recapitulate held-out annotations accumulated after 5/11/2008). 3,207 GO biological process terms are shown, grouped by annotation size at 5/11/2008 (< = 5, < = 15). The percent change in performance (median fold over random) when applying FKT compared to no FKT with each machine learning algorithm is shown for six diverse organisms. All bars are to the right of zero, indicating a performance improvement when FKT is applied for each machine learning algorithm.

We hypothesized that our transfer method could improve prediction performance for a wide range of machine learning methods. Machine learning algorithms are often based on distinct learning models and assumptions, thus any widely applicable annotation transfer method must be robust to not only the biological variability (e.g. different organisms or pathways) but also to this modeling variability. Thus in addition to SVM, we evaluated two widely used state-of-the-art learning methods: L1-regularized logistic regression [28] and Random forest [29]. We trained both classification methods with and without FKT and evaluated on the held-out set of annotations. FKT improved prediction accuracy across each machine-learning algorithm and organism (Figure 4). In particular, these improvements were consistent across biological process annotation sizes (< = 5 and < = 15). Altogether, these results indicated that FKT could recover biological processes that would be otherwise missed by most prediction methods, and that the transfer had wide applicability - improving performance across diverse organisms and machine learning algorithms.

### Genes predicted to processes with no prior annotations in the study organism reflect subsequent experimental findings

We coupled FKT with an SVM and applied the machine learning classifier to predicting novel gene functions in six organisms. These predictions included gene-process membership for 8,091 GO biological processes currently without experimental annotations in at least one organism. Supervised machine learning methods would be unable to predict novel genes to these biological processes without annotation transfer. They represent a wide range of biological pathways and processes ranging from development and metabolism to immune response and response to various stimuli (a complete list of these GO terms is in Text S2, categorization and specificity of these terms are shown in Figure S2, S3).

For example, the biological process *regulation of exit from mitosis* (GO:0007096) represents a crucial mitotic cell cycle process that enables cells to regulate their exit from M phase. This process had no experimental annotations in *Danio rerio* at the time of our study, however had been extensively studied in the model organisms *Saccharomyces cerevisiae* [32], *Mus musculus* [33] and *Drosophila melanogaster* [34]. Our functional cross-annotation method has identified a total of 18 genes in *Danio rerio* with functional analogs annotated to this process (11 from yeast, 5 fly, 1 mouse and 1 rat), enabling novel predictions of gene membership to this process.

Our top gene prediction for this process, *cdh2*, has been experimentally confirmed in a recent study examining cell cycle progression in *cdh2* mutant retina cells [35]. Interestingly, *cdh2* is not only a novel prediction in *Danio Rerio* (i.e. this gene function was unknown at the time of our study), but also no *cdh2* homologs are known to be involved in the *regulation of exit from mitosis* in other organisms. *Cdh2* is a member of the cadherin protein family, which are important transmembrane proteins that play a crucial role in cell adhesion in multi-cellular organisms. Methods that employ only sequence similarity would be unable to predict this because *cdh2* homologs have not been annotated to this process in other model organisms. Furthermore, prediction methods without FKT will miss this finding because there are no existing *Danio rerio* annotations to this process. Only methods coupling FKT with a machine learning algorithm can take advantage of information from the single cell model organism *Saccharomyces cerevisiae*, where cell-cycle checkpoints have been extensively studied [36], and successfully predict this finding in the multicellular model organism *Danio rerio*. This *in vivo* experimental result demonstrates FKT's utility for predicting novel genes to understudied processes. In addition, by coupling functional transfer to machine learning methods that leverage organism-specific functional data collections, we can make reliable gene-process predictions even without an annotated sequence-homolog.

### Cross-annotation among functional analogs improves prediction accuracy for small processes

**Figure 5:**
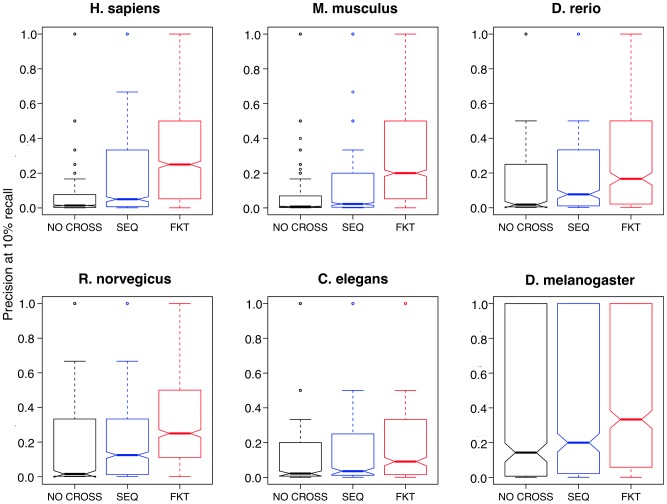
Functional knowledge transfer (FKT) improves performance for predicting small processes. The performance of two knowledge transfer methods (FKT and sequence-only) and a baseline method (with no cross-annotation) are compared. Shown here are results of threefold cross-validation for small processes (< = 15) that represent specific or understudied pathways. FKT paired prediction method shows improved performance compared to both sequence-only transfer and the baseline method.

To compare our functional transfer method, which applied a more specific annotation transfer, to commonly used methods that used only sequence homology, we evaluated a method that did not leverage functional similarity and a baseline method without any cross-annotation. In this sequence-only method, all homologous gene pairs (reciprocal BLAST best hit gene pairs) were targets for annotation transfer - any biological process annotated to a gene was transferred to its reciprocal best-hit gene in all organisms. To obtain a representative set of gene-process annotations for evaluation, we conducted a threefold cross-validation on genes that had experimental biological process annotations, and evaluated the SVM classifier prediction performance on each corresponding held-out set of biological process annotations. The results of the comparison showed that although both methods improved performance for small processes, FKT showed greater performance gains (Figure 5, evaluation results of all GO terms in Text S3). In a few organisms, the performance gains were substantial - for example, in human and mouse, the median performance (precision at 10% recall) increased more than fivefold.

Upon examining the processes that improved the most when compared to a sequence-only method, many pathways and processes with transcriptional based regulatory control showed improved performance using FKT. *Response to mechanical stimulus*, *ameboidal cell migration*, *regulation of neuron differentiation* and *striated muscle cell development* were among the top improved processes in all organisms using FKT compared to sequence-only. Unsurprisingly, these processes have been well known to be tightly regulated through transcriptional programs (e.g. stress response, developmental TF gradients) [37]–[39] and have multiple datasets measuring the transcriptional profiles incorporated in our functional networks [40]–[42].

We expect that FKT will continue to improve as the functional genomics compendia for many organisms continue to grow, including expression and other types of measurements across multiple perturbations. An additional advantage of a functional genomics similarity approach, as shown in [25], is the ability to differentiate functional differences in tissue specificity between sequence homologs. The example of mouse RNA polymerase II elongation factor *Supt5h* and its direct sequence-ortholog *C. elegans spt-5* highlight this issue. FKT determined these sequence-orthologs as not being functional analogs. Indeed, mouse Supt5h is predominantly neuronal, while *C. elegans* SPT-5 is non-neuronal and primarily expressed in the intestine and pharynx [43]–[45]. Even though these sequence-orthologs have diverged in tissue specificity, they still share high sequence similarity and a sequence-only method would inappropriately transfer all functional annotations between them.

### *In vivo* validation of *Danio rerio* gene *wnt5b* involvement in the establishment of heart asymmetry

In all vertebrates, the heart develops with a distinct left-right (L-R) asymmetry during embryonic morphogenesis. Deviations in left-right heart development can lead to complex congenital heart defects that are among the most common human neonatal diseases [46], [47]. During cardiac morphogenesis in *Danio rerio*, two distinct stages of cell migrations lead to the final asymmetries of the heart. In the first stage, called “heart jogging”, myocardial cell migration within the cardiac cone place the ventricular cells to the left side, while atrial cells remain near the midline. In the second stage of “heart looping”, the heart tube bends and forms a loop that places the ventricle to the right of the atrium. Although the steps of cell migration progression leading to left-right heart asymmetry are beginning to be explored [48]–[51], an understanding of how it is achieved mechanistically is still lacking.

In Gene Ontology, the biological process term “determination of heart left right asymmetry” (GO:0061371) represents the developmental pathways regulating heart jogging and looping. To validate our prediction method (FKT coupled with SVM), we investigated the top five predicted genes that had not already been annotated to this GO term: *sox32*, *wnt5b*, *ndr1*, *tbx1* and *lft1*. We found existing literature evidence confirming the involvement of four of the five genes (*sox32* [52]–[55], *ndr1* [56], *tbx1* [57], and *lft1* [58]–[60]). Although there existed experimental results confirming the role of these genes in influencing heart asymmetry, these results had not yet been curated by GO annotators. For example, in a knock-out experiment of our top predicted gene (*sox32*/*casanova*), *Danio rerio* embryos had fewer dorsal forerunner cells which led to defects in Kupffer's vesicle formation and subsequent left-right patterning of the heart, confirming that *sox32* was required for proper establishment of heart asymmetry. The only gene among the top five without existing experimental support was *wnt5b*, our second ranked prediction after *sox32*. Previous work had shown the involvement of *wnt5b* in cell migration during gastrulation [61] but the gene had not been specifically associated with heart left-right asymmetry regulation. To experimentally validate our prediction of *wnt5b* to left-right heart determination, we knocked down its function by means of morpholino antisense oligonucleotides (MO) [62].

**Figure 6:**
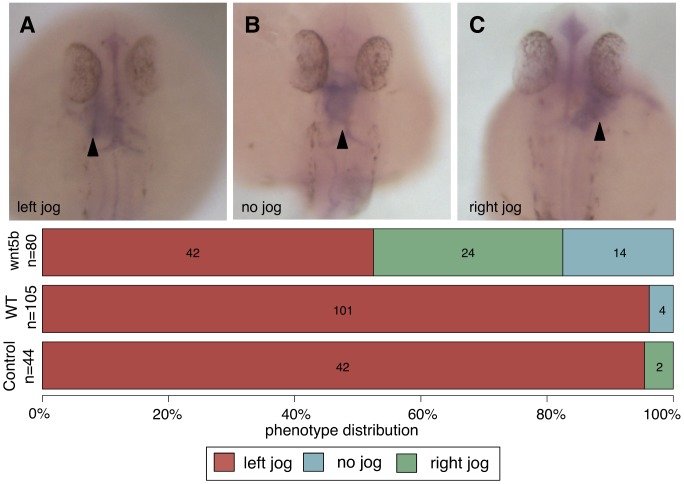
Knockdown of *wnt5b* leads to defects in zebrafish heart asymmetry. Morpholinos (MO) against *wnt5b* were injected into zebrafish embryos at the 1–2 cell stage. Embryos were evaluated for heart jogging at 27 hour post fertilization and scored as either left (C), right (B), or no jog (A). While control MO injected embryos had predominantly left-jogged hearts, embryos injected with the wnt5bMO displayed randomized heart jogging with 48% of embryos displaying right or midline jog.

A significantly greater proportion of embryos where *wnt5b* was knocked down with a morpholino (Figure 6) had a defective heart jogging phenotype (Fisher's exact test p-value<0.001). In total, 48% of morpholino treated embryos showed either right-sided heart jog or midline jog comparable to previous genes known to be involved in this biological process [63]–[65]. Only 4% of wild type and control-MO treated embryos exhibited this phenotype. This phenotype is likely due to the disruption of asymmetric expression of the TGFbeta member Nodal (data not shown), which is typically asymmetrically expressed on the left side of vertebrate embryos during somitogenesis. Left-sided Nodal in *Danio rerio* myocardial cells directs their subsequent migration during asymmetric cardiac morphogenesis [48], [51]. Further investigation would be necessary to understand the mechanistic role of *wnt5b* in left-right heart determination, however our *in vivo* experiment confirmed the regulatory role of *wnt5b* in *Danio rerio* left-right asymmetry determination in heart development, as our method predicted.

## Discussion

This study demonstrates that state-of-the-art machine learning methods coupled with our functional knowledge transfer method can accurately prioritize novel genes of understudied processes. Previous methods have focused on incorporating functional genomic data primarily as input data [66]–[69]. In contrast, here we demonstrate that the prevalence of understudied processes and the abundance of genomic data provide an opportunity to improve the accuracy of cross-organism annotation transfer and extend prediction coverage to processes with no prior annotations. We now integrate FKT into our IMP web-server [26]. This makes IMP a web interface for exploratory analysis covering all organisms included in this study across 10,653 biological processes (http://imp.princeton.edu). Functional knowledge transfer allows IMP to also include gene predictions for processes currently unannotated in an organism. Although in our current study we have experimentally followed up on our top predicted gene, all of our predictions in IMP are shown with estimated probabilities allowing biologist to draw a threshold dependent on how much the assay costs, and how important it is to find true positives (versus not finding false positives). In addition, the website includes the Bayesian functional relationship networks that were used for mapping functional analogs and used as input features to the machine learning methods. In particular, to the best of our knowledge, we include the first zebrafish (*Danio rerio*) functional relationship network.

We anticipate that our approach can be extended beyond the six organisms shown in this study, as it is especially beneficial in organisms that have high-throughput genomic data with sparse annotations (e.g. frog, slime mold). Next-generation sequencing is further increasing the diversity of organisms that are measured on the genome-scale, and functional knowledge transfer can help us understand and annotate the roles of genes in such emerging model systems. Functional knowledge transfer allows for accurate hypothesis generation and experiment guidance even for pathways with no previous experimental knowledge in a given organism, thus benefiting human biology, broadly studied organisms such as mouse and fly, and newly adopted model systems.

## Materials and Methods

We developed a functional knowledge transfer method and applied this method to predicting gene functions in six organisms using a functional network based classification strategy. In summary, data integration was performed using a regularized naive Bayes classifier, which summarized the data compendium into organism specific function relationship networks. Edges in functional relationship networks represented, given all collected data from that organism, the posterior probability of a gene pair co-functioning in the same biological process. Next, a collection of organism specific experimental annotations supplemented with cross-annotated gene annotations (based on both sequence and functional similarity) was used as gold standard for each GO biological process to train a GO term specific SVM with the functional relationship network as features. To test for robustness across different machine learning algorithms, L1-regularized logistic regression and Random forest were also evaluated by coupling both algorithms with the functional knowledge transfer method. Final predictions were made on a total of 10,653 unique biological processes. We experimentally validated our method's predictions for the determination of heart left-right asymmetry in *Danio rerio*. Of our top five predictions, four were validated via existing but un-curated experiments from the literature. We validated the fifth, *wnt5b*, using a morpholino knock-down assay.

### Integration and summary of organismal data compendia

#### Data source and pre-processing

We collected 2,444 microarray datasets from NCBI Gene Expression Omnibus (GEO) covering a total of 43,865 conditions across seven model organisms. Probes were collapsed and normalized according to the procedure described in [69] and the Fisher's z-transformed pearson correlation were calculated for each gene-pair as described in [10].

Physical and genetic interaction data from BioGRID [70], IntAct [71], Mint [72], and MIPS [73] were processed as counts of experimental assays that support an interaction between two genes (e.g. a gene pair with evidence from two-hybrid and western blot would receive two counts). Potential transcription factor (TF) to target gene associations were obtained from Yeastract [74] and TF binding site motifs retrieved from Jaspar [75]. Yeastract's predicted TF-gene relations were treated as pair-wise binary scores. For Jaspar, we searched for possible transcription factor binding sites by scanning each TF profile in 1 kb upstream sequence of all genes using FIMO [76]. Motif matches were treated as a binary score (present if p-value<.001 and not-present otherwise) and the final gene pair score was obtained by calculating the pearson correlation between the two genes' binary score vectors.

Phenotype and disease data from SGD [77], MGI [78], Wormbase [79], Flybase [80], GSEA [20], Zfin [81] were incorporated into our functional networks by summing the co-occurrences of gene pairs in all phenotypes/diseases and normalizing by the size of the phenotype/disease. For gene pair, *i, j* the scoring function is the following:
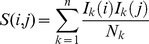
where function *I_k_(i)* and *I_k_(j)* are the indicator functions that have the value 1 when gene *i* or *j* is annotated to the phenotype or disease, *n* is the total number of phenotypes/diseases, and *N_k_* is the total number of genes associated with the phenotype or disease *k*.

Protein sequence similarity between genes was obtained from Biomart [82], and protein domain data were treated as binary evidence from PfamA [83] and Prosite [84].

#### Generating functional relationship networks

To summarize the processed heterogeneous genomic data, we generated one global functional relationship network per organism. We applied Bayesian integration, specifically a naïve Bayes classifier, to systematically deal with differences in accuracy and relevance of each data source for predicting gene functional relations. Gene pairs co-annotated to a set of 433 expert selected Gene Ontology [31] biological process fringe terms were used as known functionally related genes (i.e. positive examples) [69], [85]. Gene pairs not co-annotated to any terms in the GO fringe, KEGG [86], PID [87] or Biocyc [88] were considered as unrelated (i.e. negative examples) except in the following cases:

1. A gene pair was annotated to terms overlapping with a hypergeometric P-value below 0.05
2. A gene pair was annotated to a set of ‘negative’ GO terms that define minimal relatedness (as described in [69])

If a gene pair met either of the two conditions, it was excluded from unrelated pair generation (i.e., they were neither related nor unrelated for training). Thus this formed a set of global related and unrelated gene pairs to be used for training and evaluation.

One binary regularized naive Bayes classifier was trained per Gene Ontology fringe term (i.e. biological process/context). Each naive Bayes classifier contains one class node determining the membership of a gene-pair to the biological process and organism specific dataset nodes conditioned on the class node. When integrating large number of genomic datasets, the naive Bayes assumption of conditional independence among datasets can no longer be justified. We have shown that a mutual information based parameter regularization for naive Bayes classifiers can alleviate the conditional dependency among datasets [69]. In this work, we make modifications to our prior method by directly estimating the conditional dependency between a dataset by limiting the mutual information calculation between datasets to gene-pairs that are not functionally related. This heuristic enables us to estimate the conditional dependency between datasets without having to regress out the incomplete functional relation class node information. Specifically, the heuristic sum of shared information *U_k_* is now:
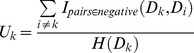


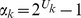
where *I_pairs_*_∈*negatives*_ is the mutual information between dataset *D_k_* and *D*_i_ among gene pairs not known to have a functional relationship (i.e. negative gene pair examples) and *H* is the single dataset entropy. Then we use the exponential decreased ratio (α_k_) to weight a given dataset's likelihood function. Finally, the naive Bayes functional relationship posterior probability for gene pair *i,j* is the following:
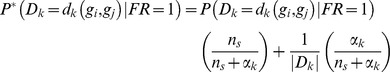


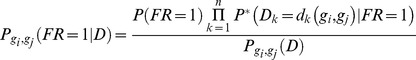
where the weighted dataset likelihood function is P^*^, *d_k_(g_i_, g_j_)* is the experimental value for gene pair *i,j*, |*D_k_*| is the total number of discretization levels and *n_s_* is a pseudocount set to 3 in our integration based on cross-validation results.

Finally, with biological process specific functional relation networks predicted by each naive Bayes classifier, we averaged the edge probabilities from each process specific functional network to generate the final global functional relationship network.

### GO biological process gold standard construction through cross-annotation

In total, 10,653 GO biological process terms were predicted for new gene annotations covering six organisms, *Homo sapiens*, *Mus musculus*, *Rattus novegicus*, *Drosophila melanogaster*, *Danio rerio* and *Caenorhabditis elegans.* We limited the positive examples for each GO term to propagated experimental GO annotations with GO evidence codes EXP, IDA, IPI, IMP, IGI and IEP (all “NOT” annotations were removed). In addition, to leverage the research strengths across organisms, we transferred gene annotations among six organisms plus yeast, first based on sequence similarity and second filtered by function similarity. In detail, we start with all sequence paralog and ortholog gene relations within each TreeFam [22] gene family. Next, based on our previous algorithm [25], we filtered for functional analogs among all paralog and ortholog gene pairs using our functional relationship networks. We define a functional analog to be a gene pair that has a significant number of overlapping TreeFam gene families among its closest gene neighbors in the global functional relationship network (a functional network is converted into a binary network by using a probability cutoff of 0.5). We defined a gene pair's score as the following:
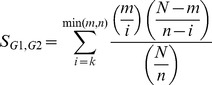
where *m* and *n* are the number of TreeFam gene families in each gene *G1* and *G2*'s direct neighborhood in the functional network, *k* is the number of overlapping TreeFam gene families between gene *G1* and *G2* gene neighbors and *N* is the total number of TreeFam gene families around gene *G1* and *G2*. The functional similarity score is the probability of observing greater or equal to the number of overlapping gene families by chance, thus can be interpreted as a hypergeometric p-value. We used a score cutoff of < = 0.01 to consider a gene pair as functional analogs.

Finally, all experimental annotations are propagated between functional analogs. In total, our supervised functional knowledge transfer allowed us to make predictions for 8,091 additional GO biological processes, thus extending our predictions beyond simply well-studied and well annotated processes and pathways.

### Biological process prediction with network based SVM

We used the augmented gold standard genes by functional knowledge transfer and functional relation network as features into state-of-art machine learning algorithm Support Vector Machine (SVM) to predict novel biological process gene annotations. Our functional relation network based SVM method has shown to outperform methods that directly input the raw data into the SVM or a simplistic sum of the functional networks to the positive examples [89].

For each biological process, the feature space was constructed as the weights in the functional relation network. Thus for each gene example, all gene edge weights connecting to the example gene were used to create the feature vector. Therefore, each organism feature count will be equal to the number of genes in the organism. The set of feature vectors for training examples were used to train a linear SVM according to the standard formulation:
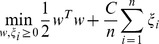


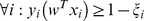
where *n* is the of training example genes, *w* is the gene weight vector, *y_i_* is the training label of gene *i* and *x_i_* is the edge weight vector connecting gene *i* to all genes in the functional network.

Finally, the unbounded SVM prediction scores were transformed into probabilities based on a maximum likelihood sigmoid fit to the SVM outputs [90].

### Additional machine learning algorithms

To validate that the observed performance improvement was not specific to any single learning algorithm, we applied the functional knowledge transfer to two additional widely used machine learning methods: L1-regularized logistic regression and Random forest. Regression analysis coupled with regularization has been a broadly used approach to control for the bias-variance trade-off [91]. In particular, L1-regularization has been successfully used in many methods for shrinkage and feature selection applications, most famously in the works of LASSO [92]. By coupling L1-regularization with a logit link function, conditional probabilities of a gene membership to a biological process can be computed based on selected genes of predictive power. The predictive gene weight vector *w* was obtained by the following regression problem:
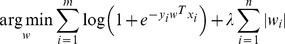
where λ>0 is the regularization parameter, *y_i_* is the training label of gene *i* and *x_i_* is the edge weight vector connecting gene *i* to all genes in the functional network.

Random forest classifiers are a combination of decision trees that are aggregated to make a final prediction. Random forest algorithms have been shown to produce improved prediction accuracy compared to a single decision tree by better estimating the contribution of each predictor through random sampling [29]. In genomic applications, Random forest has gained interest due to the many high-dimensional genomic learning problems [93]. Formally, random forest is defined by the following:
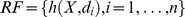
where the random forest *RF* is a set of 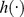 decision tree functions, trained on training examples *X* and a bootstrap sample *d_i_* from the original feature space of *D*. For classification, the votes of each *n* decision trees are averaged as shown in the following:
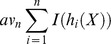
where 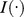 is the indicator function for the prediction class of interest. In our study, for each GO term 61 decision trees were trained on independent bootstrap samples of our original genomic training data.

### Performance evaluation

For performance evaluations for GO terms with no prior annotation, we used a three-year temporal holdout set of gene annotations for each GO biological process (one-year holdout results shown in supplemental material). The held-out gene annotations were preserved throughout the prediction pipeline (functional network integration and SVM predictions) to avoid any overestimation of performance. Although we train our SVM classifiers using the augmented cross-annotated gold standard, only the non-transferred experimental GO term annotations were used for evaluation with all transferred annotations excluded.

The GO gene association files used to create our gold standard was downloaded from Gene Ontology [31] on 5/11/2011 (all dates in mm/dd/yyyy format). To generate an accurate temporal three-year holdout we downloaded the GO gene association version archived at 5/11/2008. All annotations were propagated and only experimental examples newly annotated after 5/11/2008 for each GO term was used in the temporal evaluation. Accordingly, any GO term that had no gene annotations on 5/11/2008, but subsequently accumulated new annotations were used to evaluate our performance in predicting terms with no-known prior annotations.

To compare performance between knowledge transfer methods, we conducted an evaluation by performing a threefold cross-validation among genes that had experimental biological process annotations. This set of evaluation annotations represents a random sampling of the current knowledge base as of 5/11/2011. Identical to our temporal holdout, all evaluation annotations for each holdout were withheld from our prediction pipeline to avoid any performance over-estimation.

### Implementation

All software used in this study has been implemented in the open source and publicly available Sleipnir library [94] available from http://libsleipnir.bitbucket.org, which interfaces with the SVMperf library [95] for linear kernel SVM classifiers (the error parameter C was set to 100 for these experiments through cross-validation). L1-regularized logistic regression used the LIBLINEAR [28] and Random forest used the MILK (Machine Learning Toolkit) python package implementation with 61 decision trees per GO term.

### Morpholino microinjections and scoring of heart left-right asymmetry

The *wnt5b* morpholino (MO) and standard control MO were purchased from GeneTools. The sequence of the *wnt5b* MO used is as follows: 5′-GTCCTTGGTTCATTCTCACATCCAT-3′. Morpholinos were injected at a concentration of 6 ng/uL into the yolk of one-cell stage embryos for whole knockdown in the embryonic cells. Initial assessment (Figure 6) was performed via *in situ* hybridizations on fixed embryos using the standard protocol [96] with *cmlc2/myl7* used as a probe. Images were captured at 4× or 10× magnification using a ProgressC14 digital camera (Jenoptik) on a Leica MZFLIII microscope.

Heart laterality for each treatment (*wnt5b* MO, control MO, wild type) was evaluated in live Tg(*cmlc2::GFP*) embryos at 27 hours post fertilization. Embryos were scored as left/right/no jog based on expression of GFP driven by *cmlc2*'s heart specific promoter using a Leica SP5 confocal microscope (Figure S4).

## Supporting Information

Figure S1 **FKT cross annotation allows accurate recovery of small and unannotated terms in 1 year temporal holdout (pink: FKT+SVM, gray: SVM).** All annotations accumulated after 5/11/2010 are held out from our prediction pipeline (as outlined in Figure 2) and are used to evaluate the predictive power of FKT derived cross annotations (3 year temporal holdout is shown in main text figure 3). GO biological processes terms that acquired new annotations subsequent to our holdout date are grouped by organism and by the number of annotations at 5/11/2010 (zero, < = 5, < = 15, all). Performances at recapitulating future annotations are compared for a machine learning method (SVM) without (gray) and with (pink) learning on functional knowledge transfer (FKT) derived examples. For processes with zero annotations before 5/11/2010, no predictions can be made without cross-annotation (shown as absent performance bar).
(PDF)

Figure S2 **The categorization of newly predicted biological processes.** In total 8,091 GO biological processes without prior experimental annotation were predicted for novel gene-pathway membership by deploying FKT across our six metazoan organisms (*Homo sapiens*, *Mus musculus*, *Rattus novegicus*, *Drosophila melanogaster*, *Danio rerio* and *Caenorhabditis elegans*). Here we show the nature of these newly predicted biological process terms grouped by each process' parent term in the gene ontology (1 level in the biological process name space).
(PDF)

Figure S3 **Specificity of newly predicted biological processes.** Here we plot the specificity of 8,091 newly predicted GO biological processes without prior experimental annotations. As an imperfect proxy for biological specificity we use the depth of each process term in the gene ontology biological process name space. As examples of terms for a given depth, depth 2 leukocyte proliferation, depth 4 glomerulus vasculature development, depth 6 intermediate filament cytoskeleton organization, depth 8 purine ribonucleotide biosynthetic process, depth 10 regulation of insulin secretion involved in cellular response to glucose stimulus, depth 12 negative regulation of histone h3 k9 methylation.
(PDF)

Figure S4 **Zebrafish wnt5b knockdown in live embryos show significant deviation from wild type heart jogging.** Heart laterality for each treatment (wnt5b MO, control MO, wild type) was evaluated in live embryos at 27 hours post fertilization. Embryos were scored as left (C), right (B), or no jog (A) based on the expression of GFP driven by cmlc2's heart specific promoter. In total, 48% of morpholino treated embryos showed either right-sided heart jog or midline/no jog. Only 4% of wild type and control-MO treated embryos exhibited this phenotype. *In situ* results are shown in Figure 6 in the manuscript.
(PDF)

Text S1 **GO terms relevant in mammals (mouse, human, rat) but missing in at least one organism.**
(TXT)

Text S2 **GO terms with no experimental annotations but gene prediction enabled by FKT.**
(TXT)

Text S3 **All GO terms prediction evaluation results for temporal and random holdout.**
(TXT)
