# Supplementary figures and images for: Functional Knowledge Transfer for High-accuracy Prediction of Under-studied Biological Processes
Source: PLoS Comput Biol. 2013 Mar 14;9(3):e1002957. doi: 10.1371/journal.pcbi.1002957 (PMC3597527; doi:10.1371/journal.pcbi.1002957)

**H. sapiens**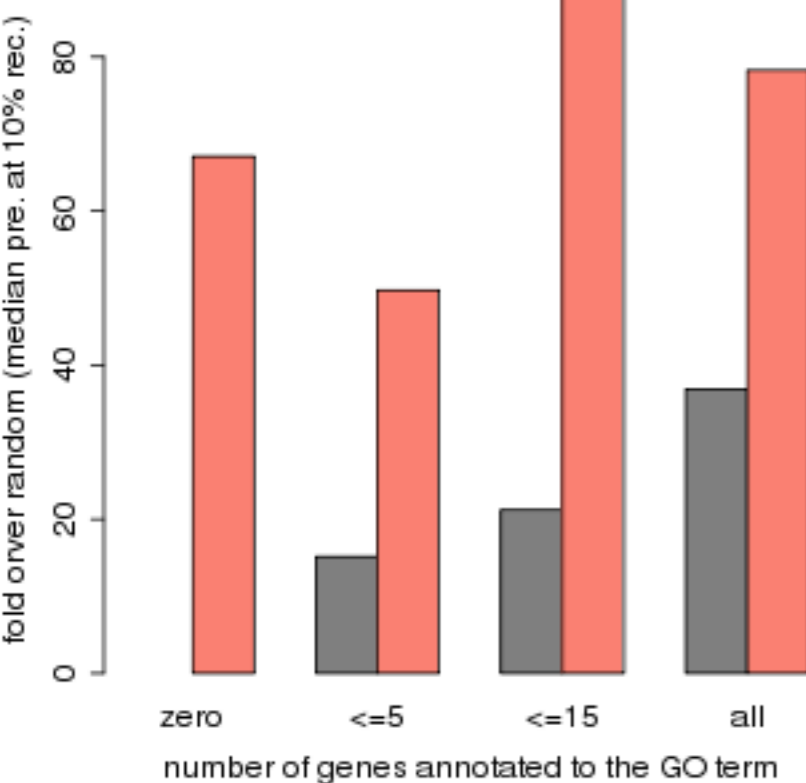**M. musculus**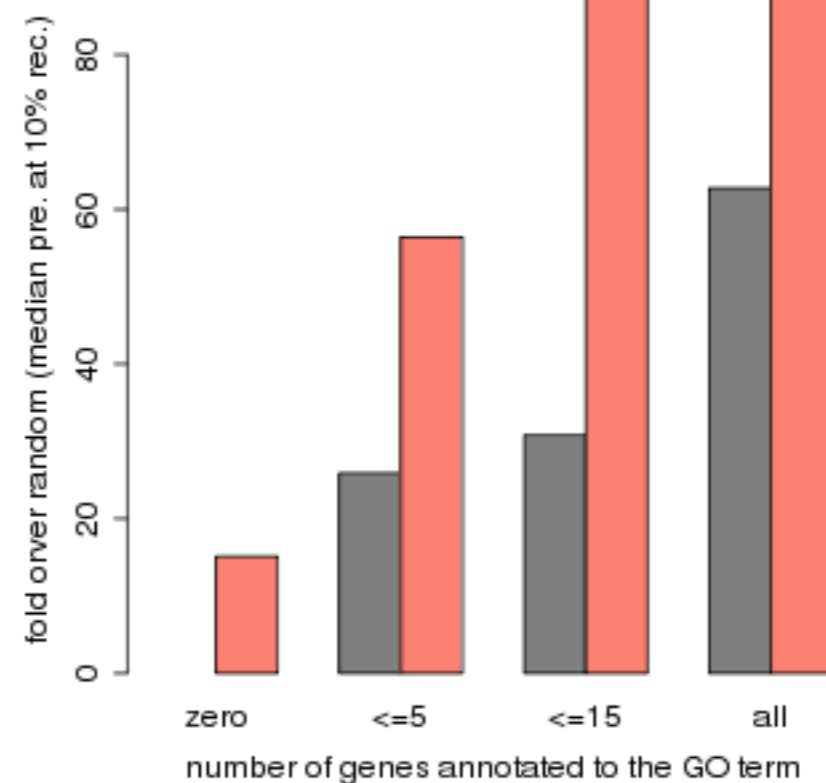**D. rerio**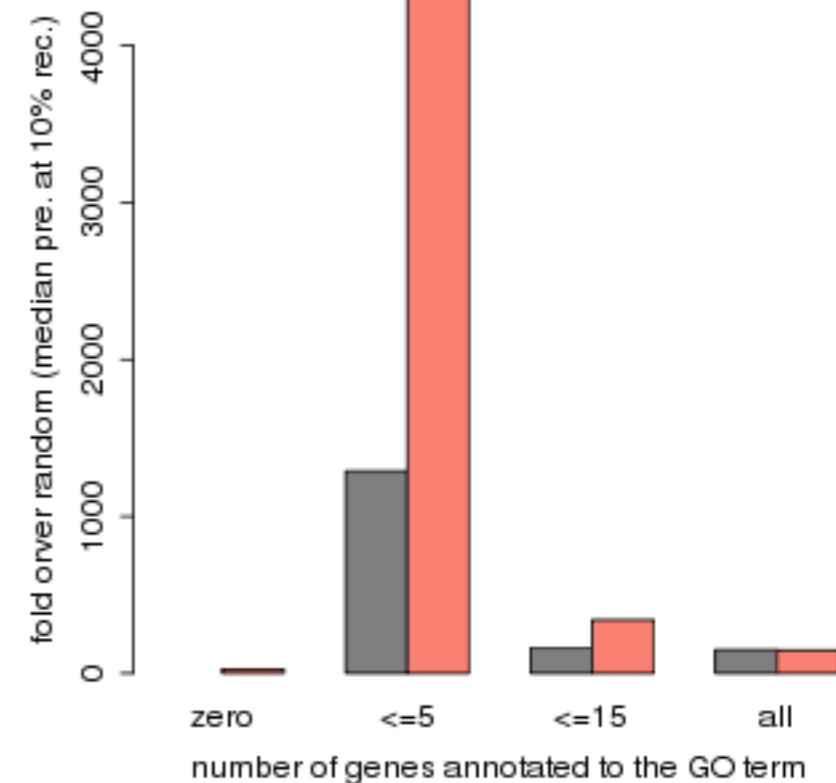**R. norvegicus**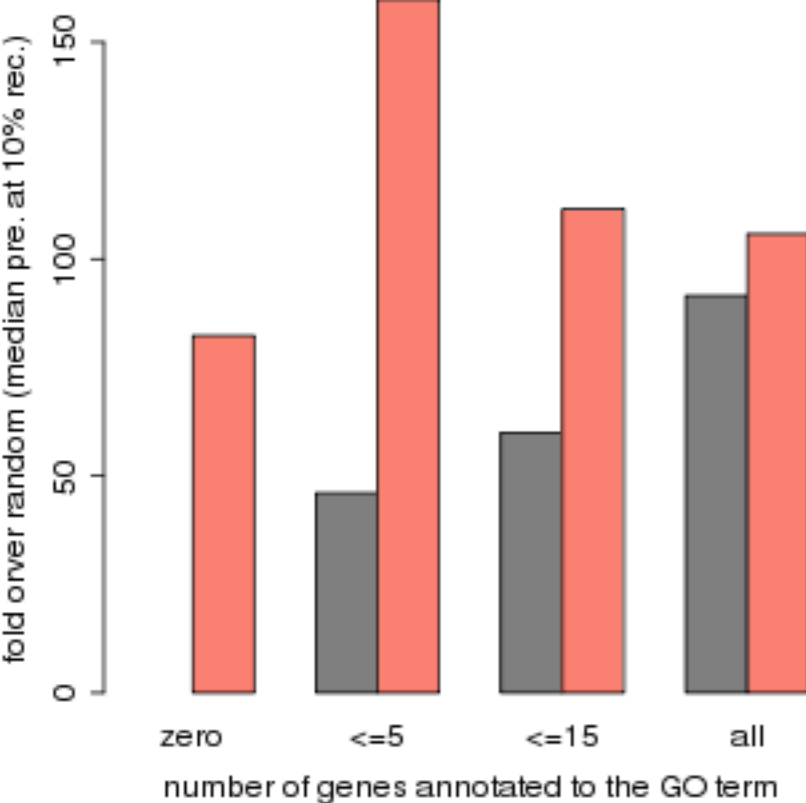**C. elegans**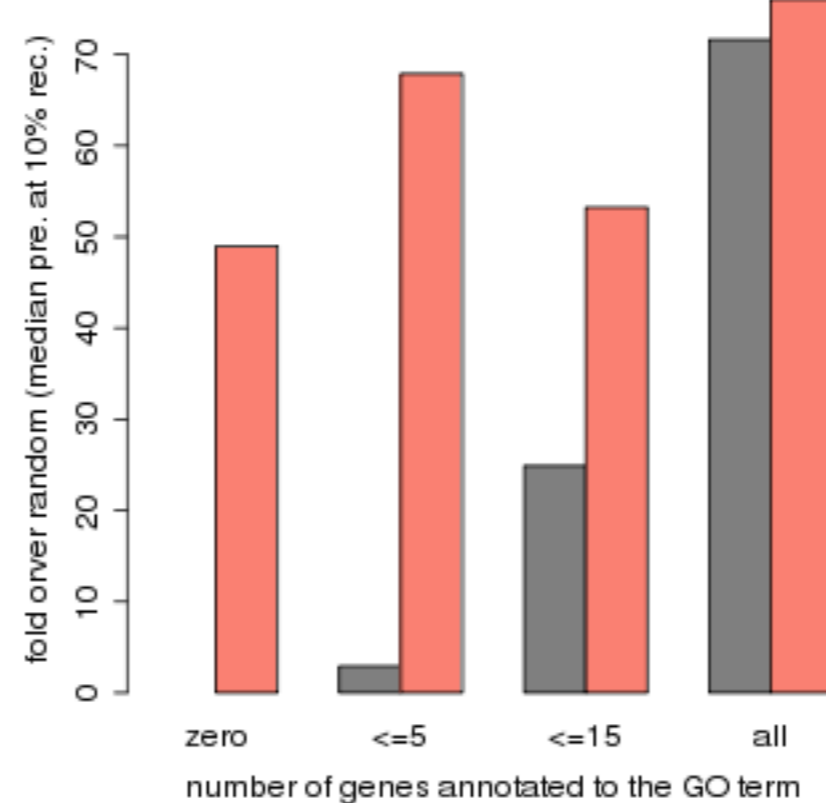**D. melanogaster**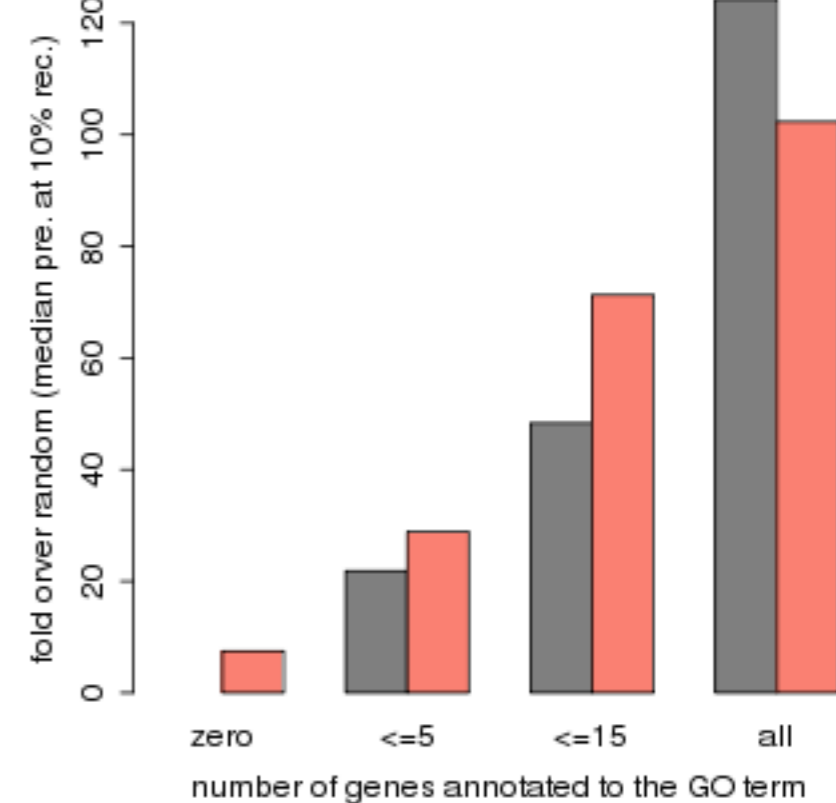

Supplement: Figure S1 — FKT cross annotation allows accurate recovery of small and unannotated terms in 1 year temporal holdout (pink: FKT+SVM, gray: SVM). All annotations accumulated after 5/11/2010 are held out from our prediction pipeline (as outlined in Figure 2) and are used to evaluate the predictive power of FKT derived cross annotations (3 year temporal holdout is shown in main text figure 3). GO biological processes terms that acquired new annotations subsequent to our holdout date are grouped by organism and by the number of annotations at 5/11/2010 (zero, < = 5, < = 15, all). Performances at recapitulating future annotations are compared for a machine learning method (SVM) without (gray) and with (pink) learning on functional knowledge transfer (FKT) derived examples. For processes with zero annotations before 5/11/2010, no predictions can be made without cross-annotation (shown as absent performance bar). (PDF) [file pcbi.1002957.s001.pdf]

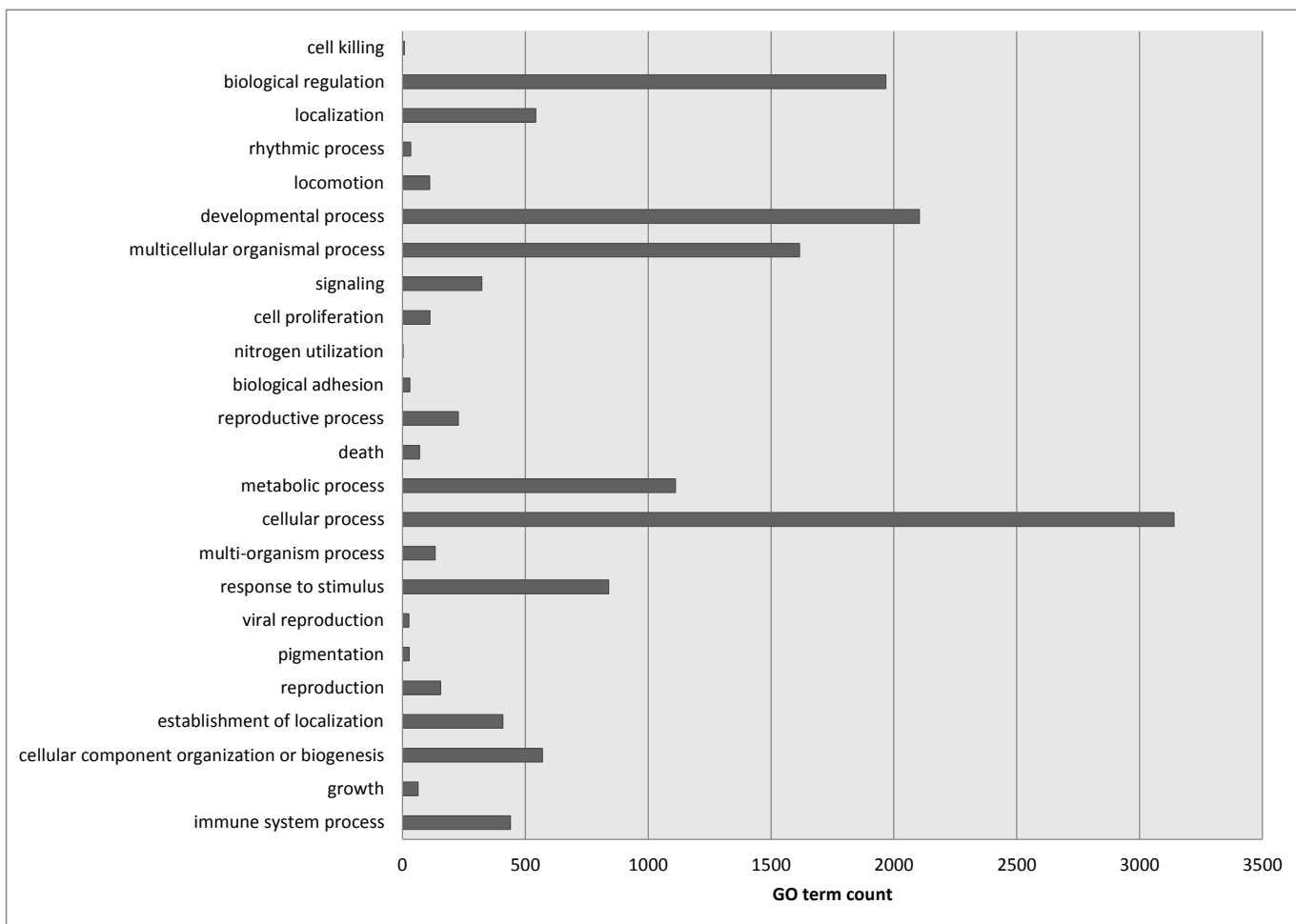

Supplement: Figure S2 — The categorization of newly predicted biological processes. In total 8,091 GO biological processes without prior experimental annotation were predicted for novel gene-pathway membership by deploying FKT across our six metazoan organisms (Homo sapiens, Mus musculus, Rattus novegicus, Drosophila melanogaster, Danio rerio and Caenorhabditis elegans). Here we show the nature of these newly predicted biological process terms grouped by each process' parent term in the gene ontology (1 level in the biological process name space). (PDF) [file pcbi.1002957.s002.pdf]

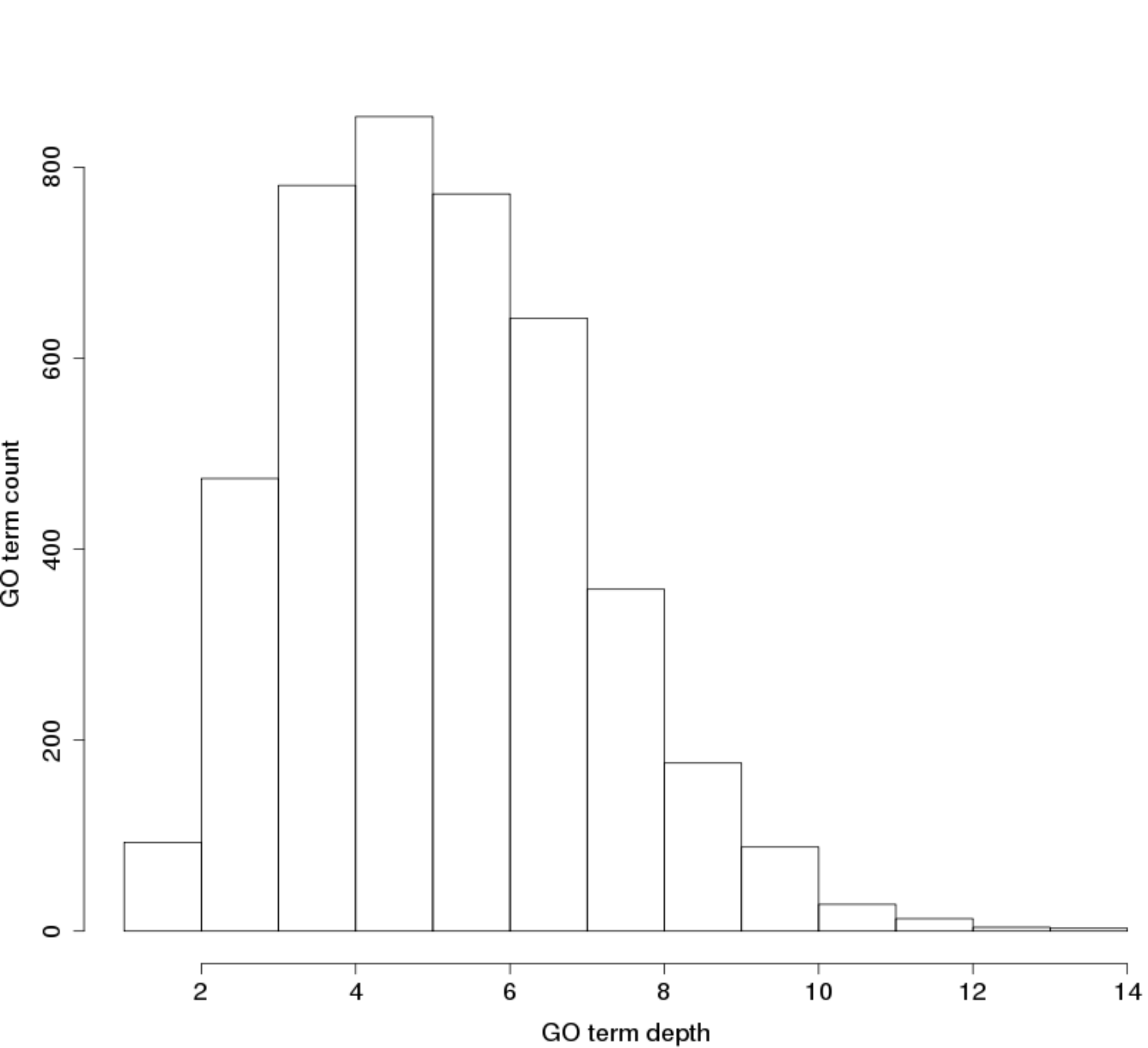

Supplement: Figure S3 — Specificity of newly predicted biological processes. Here we plot the specificity of 8,091 newly predicted GO biological processes without prior experimental annotations. As an imperfect proxy for biological specificity we use the depth of each process term in the gene ontology biological process name space. As examples of terms for a given depth, depth 2 leukocyte proliferation, depth 4 glomerulus vasculature development, depth 6 intermediate filament cytoskeleton organization, depth 8 purine ribonucleotide biosynthetic process, depth 10 regulation of insulin secretion involved in cellular response to glucose stimulus, depth 12 negative regulation of histone h3 k9 methylation. (PDF) [file pcbi.1002957.s003.pdf]

A

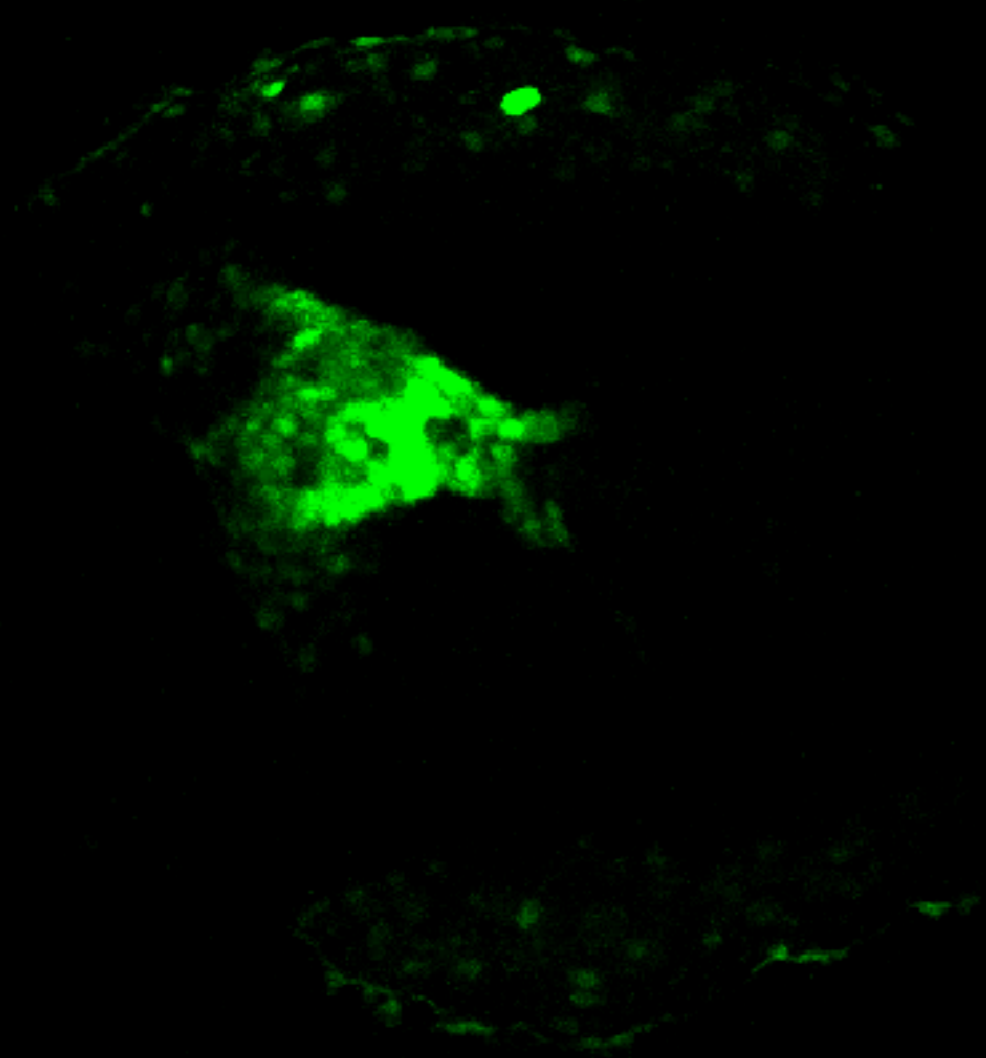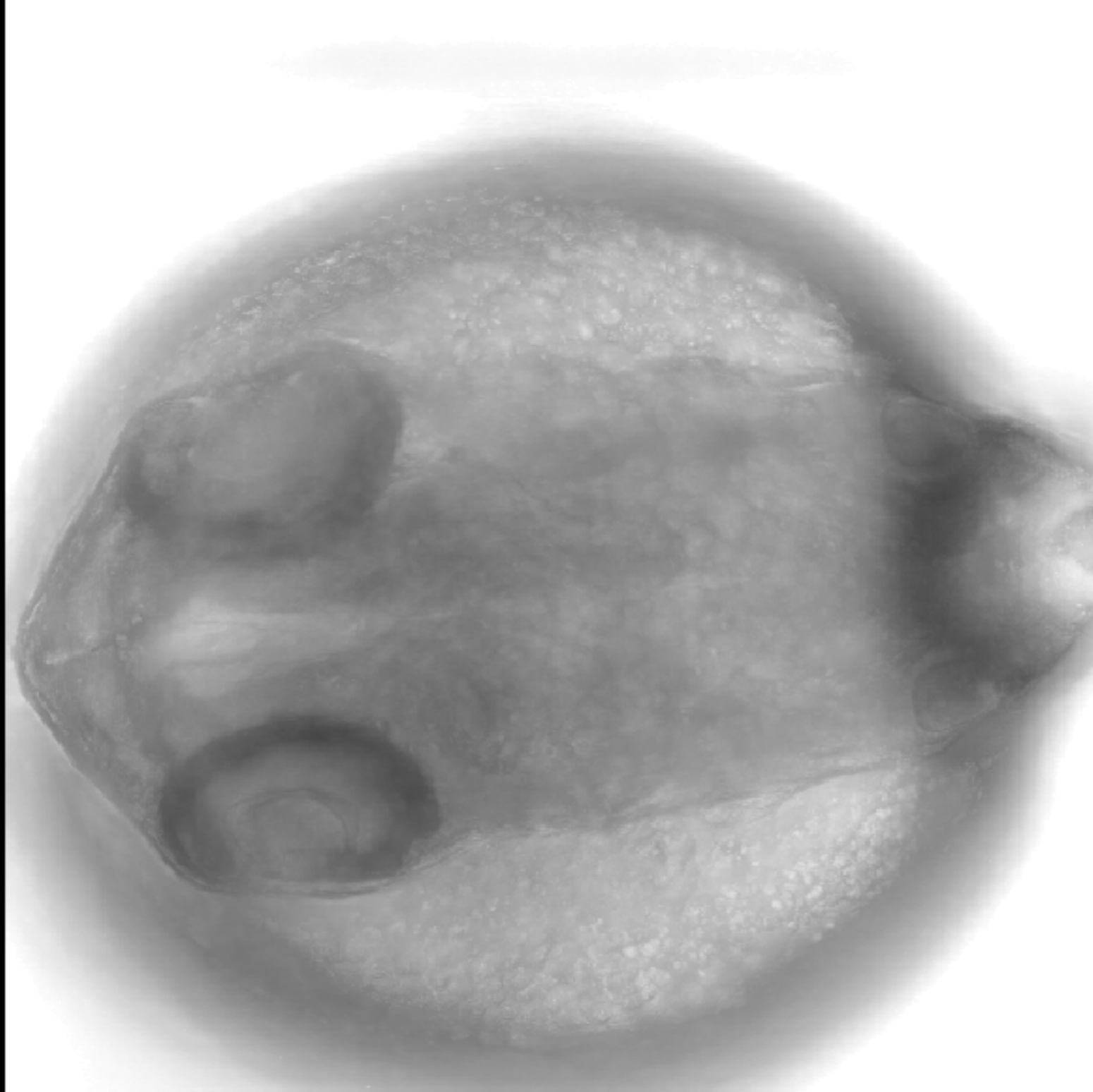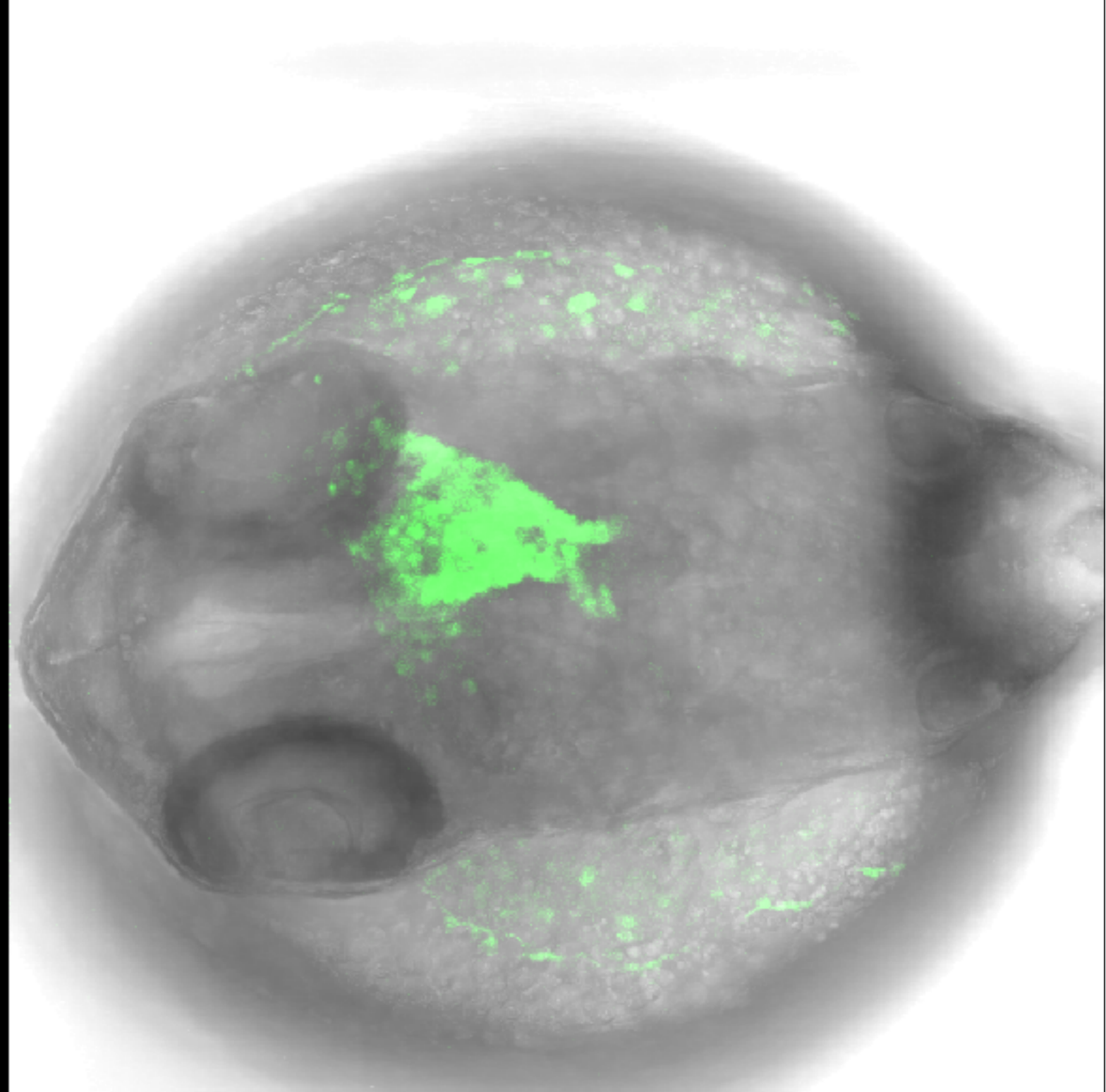

B

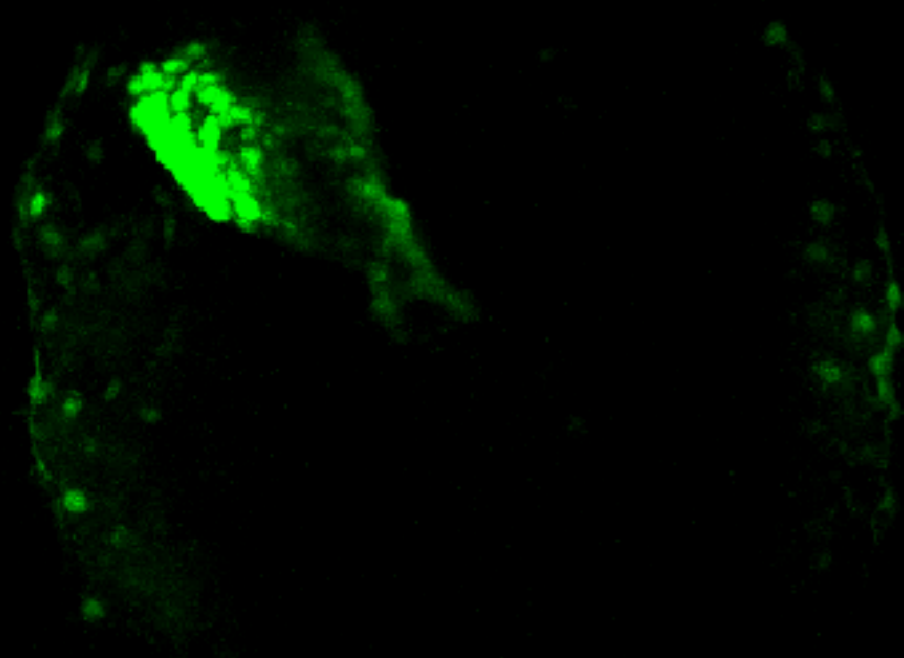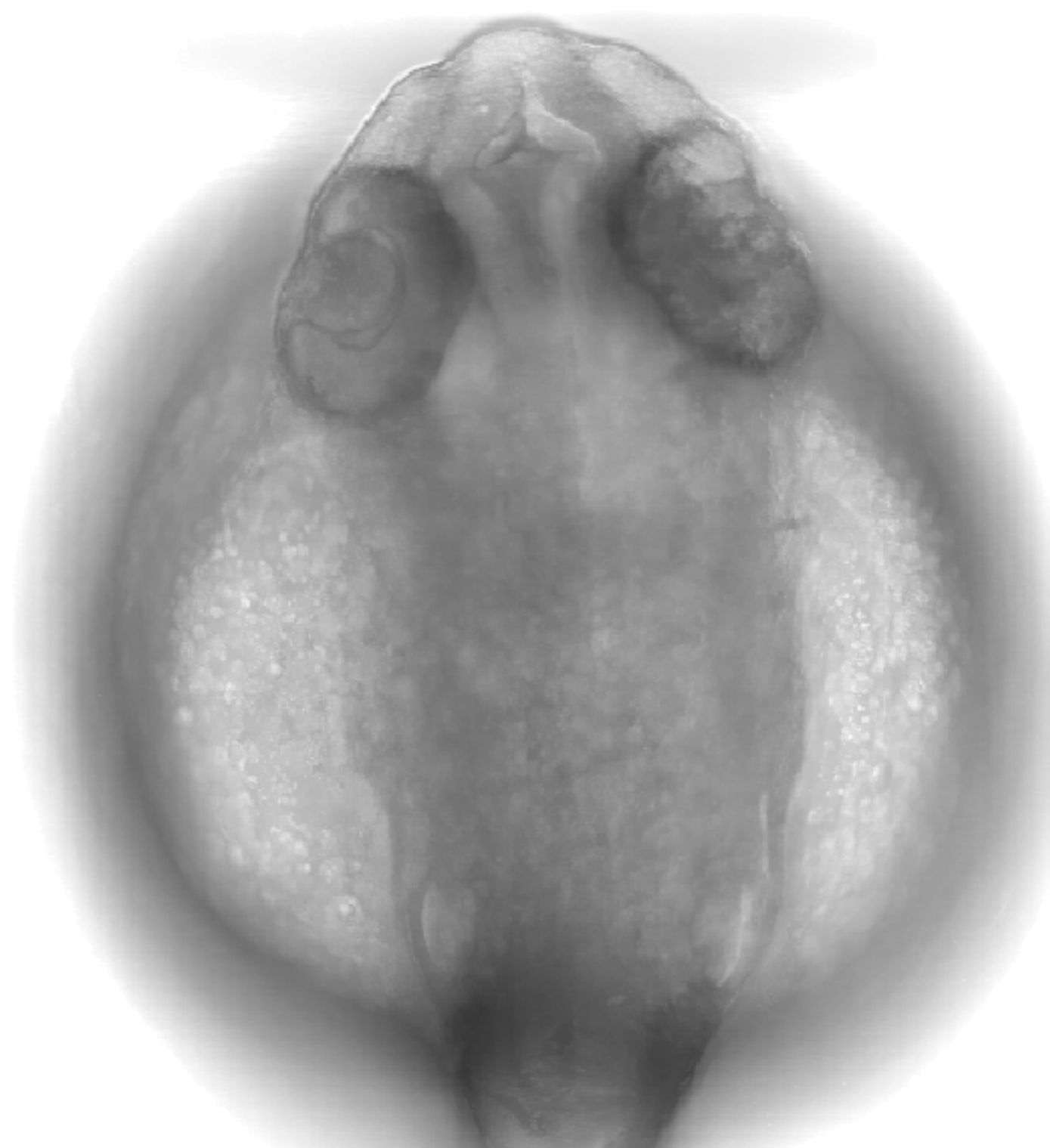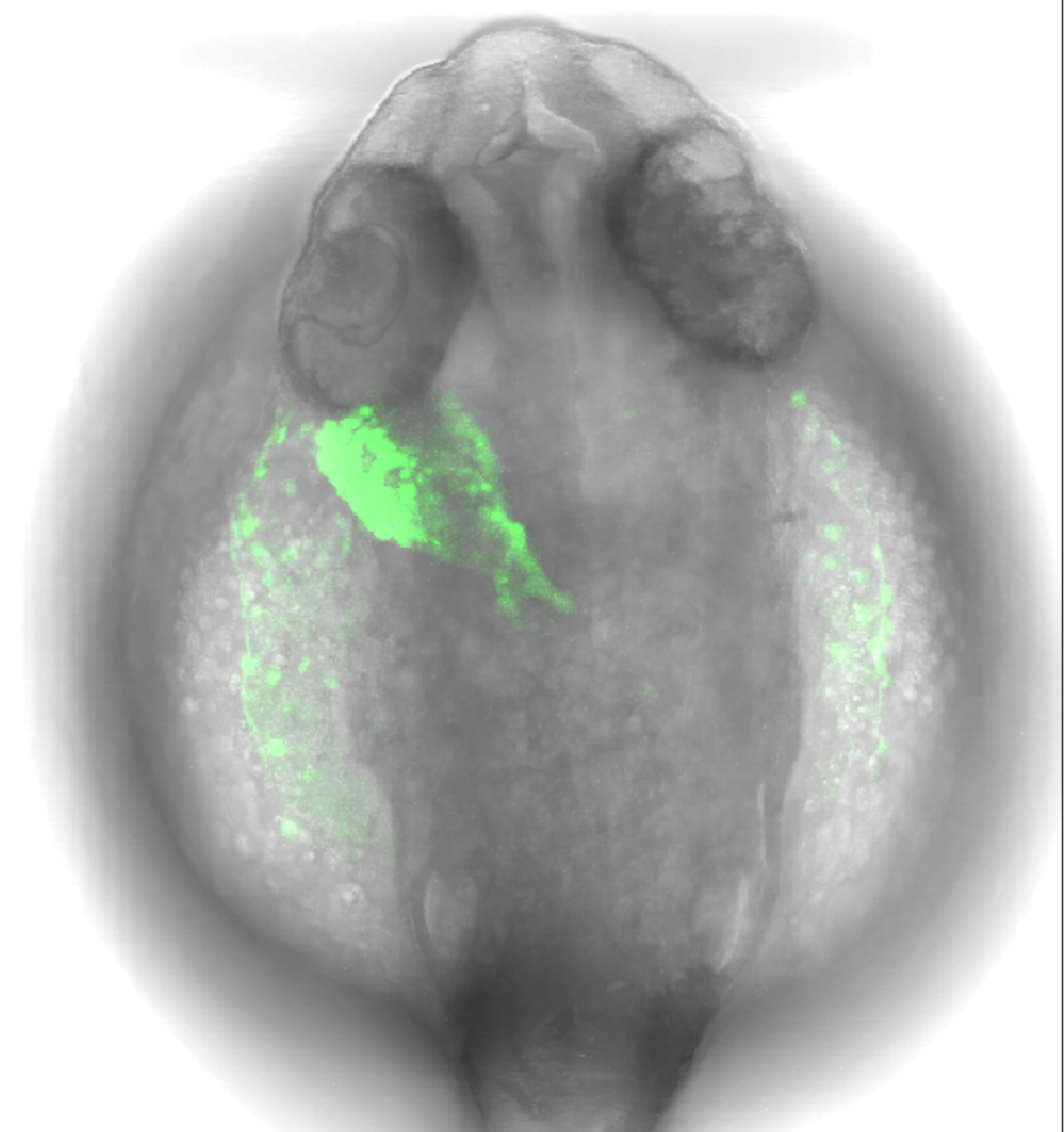

C

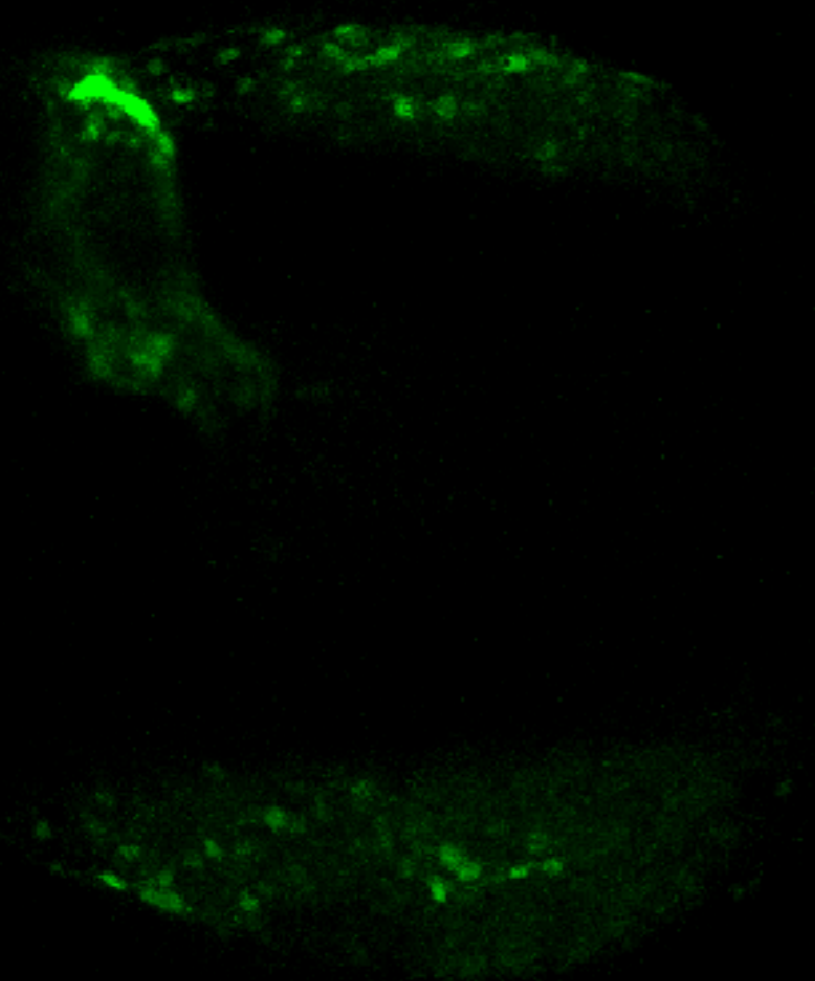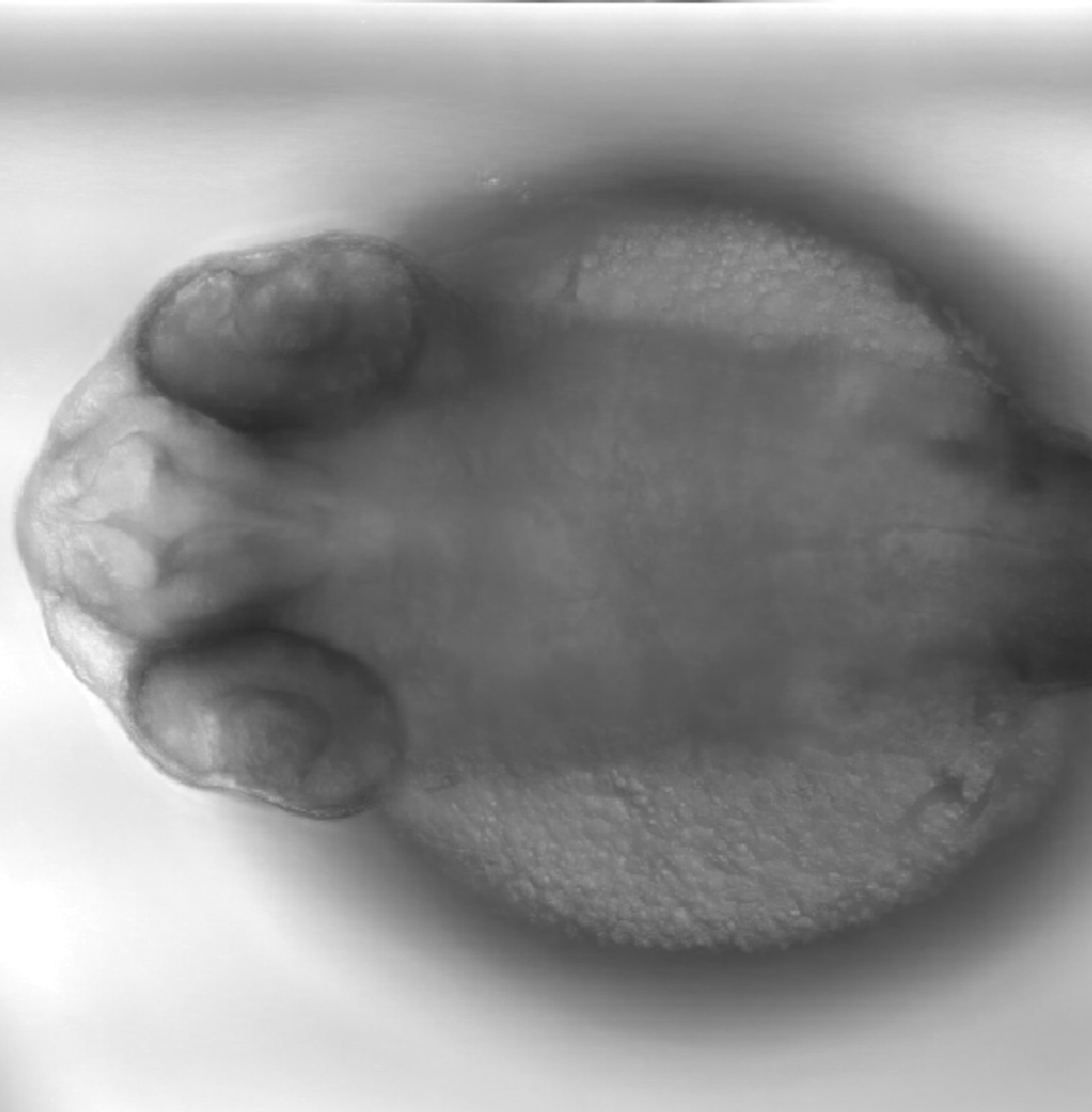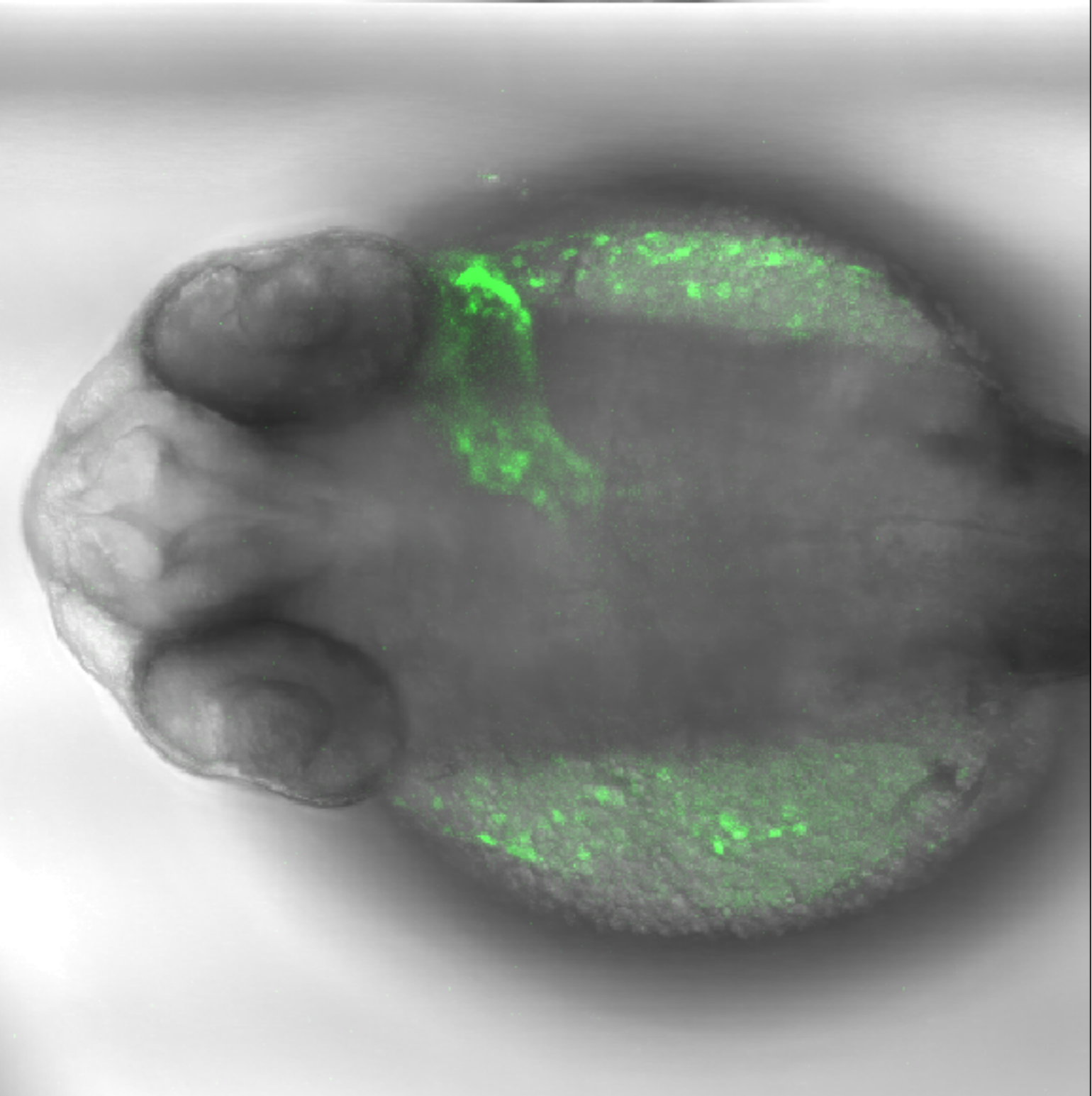

Supplement: Figure S4 — Zebrafish wnt5b knockdown in live embryos show significant deviation from wild type heart jogging. Heart laterality for each treatment (wnt5b MO, control MO, wild type) was evaluated in live embryos at 27 hours post fertilization. Embryos were scored as left (C), right (B), or no jog (A) based on the expression of GFP driven by cmlc2's heart specific promoter. In total, 48% of morpholino treated embryos showed either right-sided heart jog or midline/no jog. Only 4% of wild type and control-MO treated embryos exhibited this phenotype. In situ results are shown in Figure 6 in the manuscript. (PDF) [file pcbi.1002957.s004.pdf]
